# Annotated Bioinformatic Pipelines for Phylogenomic Placement of Mitochondrial Genomes

**DOI:** 10.21769/BioProtoc.5232

**Published:** 2025-03-05

**Authors:** Jessica C. Winn, Aletta E. Bester-Van Der Merwe, Simo N. Maduna

**Affiliations:** 1 Molecular Breeding and Biodiversity Group, Department of Genetics, Stellenbosch University, Stellenbosch, Western Cape, South Africa; 2 Department of Ecosystems in the Barents Region, Svanhovd Research Station, Norwegian Institute of Bioeconomy Research, Svanvik, Norway

**Keywords:** Taxonomy, Molecular systematics, Multispecies coalescence, Site heterogeneity, Incomplete lineage sorting

## Abstract

The limited standards for the rigorous and objective use of mitochondrial genomes (mitogenomes) can lead to uncertainties regarding the phylogenetic relationships of taxa under varying evolutionary constraints. The mitogenome exhibits heterogeneity in base composition, and evolutionary rates may vary across different regions, which can cause empirical data to violate assumptions of the applied evolutionary models. Consequently, the unique evolutionary signatures of the dataset must be carefully evaluated before selecting an appropriate approach for phylogenomic inference. Here, we present the bioinformatic pipeline and code used to expand the mitogenome phylogeny of the order Carcharhiniformes (groundsharks), with a focus on houndsharks (Chondrichthyes: Triakidae). We present a rigorous approach for addressing difficult-to-resolve phylogenies, incorporating multi-species coalescent modelling (MSCM) to address gene/species tree discordance. The protocol describes carefully designed approaches for preparing alignments, partitioning datasets, assigning models of evolution, inferring phylogenies based on traditional site-homogenous concatenation approaches as well as under multispecies coalescent and site heterogenous models, and generating statistical data for comparison of different topological outcomes. The datasets required to run our analyses are available on GitHub and Dryad repositories.

Key features

• An extensive statistical framework to conduct model selection and data partitioning and tackle difficult-to-resolve phylogenies.

• Instructions for generating statistical data for comparison of different topological outcomes.

• Tips for selecting mitochondrial phylogenomic (mitophylogenomic) approaches to suit unique datasets.

• Access to the scripts, data files, and pipelines used to enable replication of all analyses.

## Graphical overview


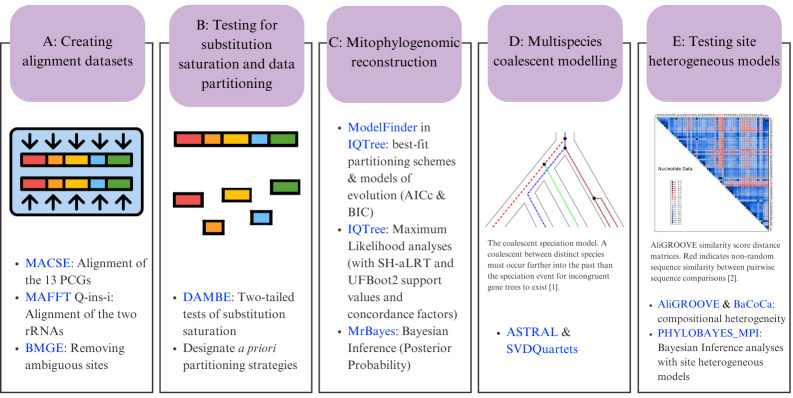


**Bioinformatic workflow for phylogenomic reconstruction using mitochondrial genomes.** In section D, the image shows the coalescent speciation model [1]; section E shows an example of sequence heterogeneity in pairwise sequence comparisons [2]. Software programs are indicated in blue.

## Background

Phylogenetics, the study of the evolutionary relationship between organisms, has gained momentum following the genomics revolution, which has allowed for the accumulation of high-quality mitochondrial genomes (mitogenomes) [3–6]. However, even with larger datasets, we are still left with inconsistencies in the phylogenetic placement of many groups, from genera to orders [7,8]. Consequently, although it is vital to ensure taxa are adequately represented [9,10], phylogenetic analysis methods are often more integral to the interpretations of hypotheses than simply accumulating more data [7,11].

Traditional mitophylogenomic studies partition concatenated alignments based on sequence properties such as gene boundaries and codon locations before selecting and optimising substitution models for each partition [12–16]. There is heterogeneity in base composition and evolutionary rates at different scales across the mitogenome [17,18], so the empirical data may violate the assumptions of the applied models of evolution. Consequently, tools like ModelFinder have been exploited to select substitution models that best fit predefined subsets of a given dataset according to a chosen statistical criterion under a maximum likelihood (ML) framework [14,16,19]. However, the a priori partitioning strategy fed into the software is often selected without accounting for the unique evolutionary signatures of a dataset [14,16,20,21]. On the one hand, compositional heterogeneity, substitution saturation, branch length heterogeneity, and incomplete lineage sorting can lead to model violations and profoundly impact phylogenetic outcomes [22–26]. On the other hand, using phylogenetic strategies that account for these factors without first testing for their presence can also be detrimental to accurate tree construction. Overpartitioning the dataset or incorrectly using site-specific models can lead to “overparameterisation” of model parameters, yielding well-supported but erroneous nodes in the tree [23,27–29].

**Figure 1.**
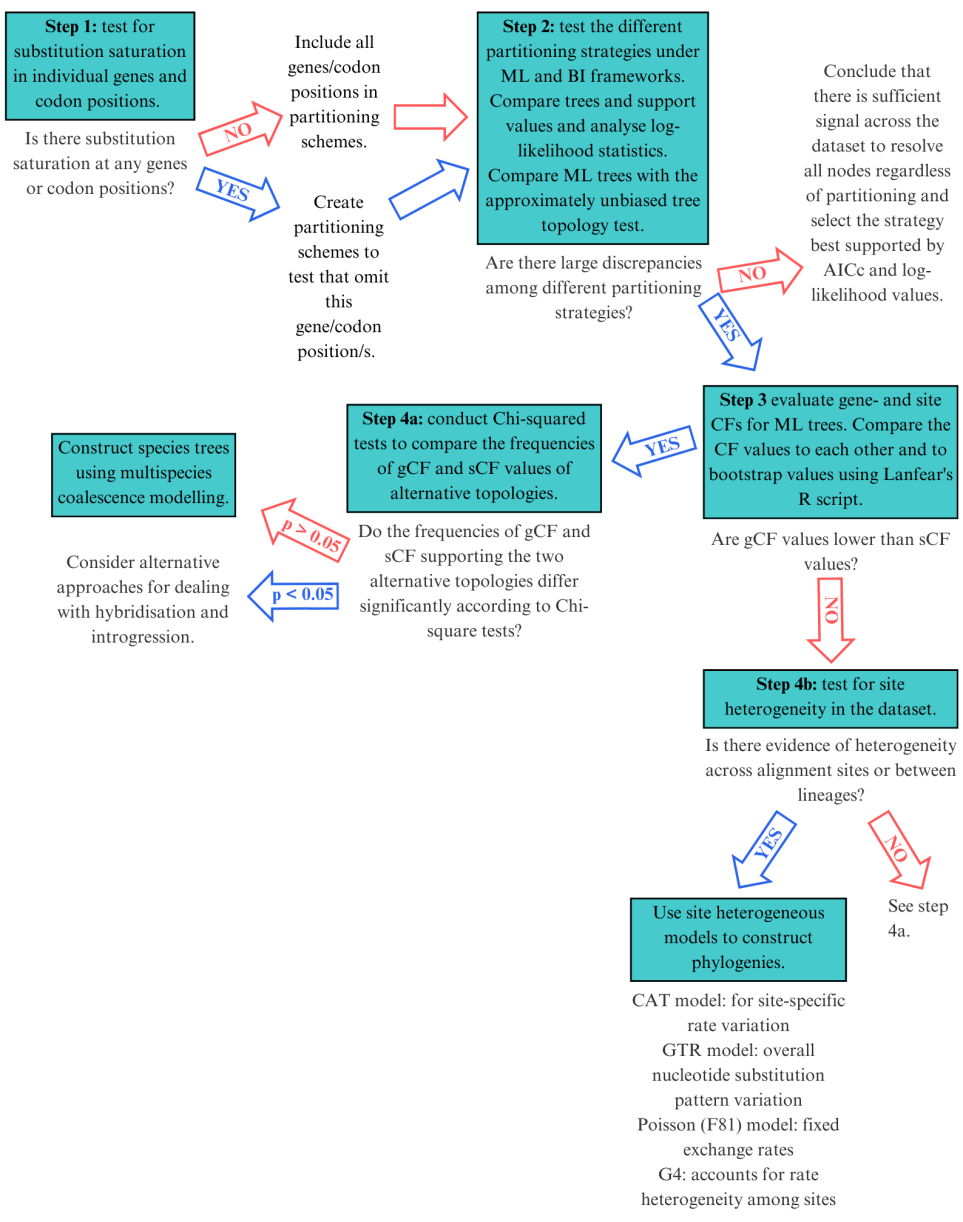
Selecting the right mitophylogenomic approach for your dataset. ML: maximum likelihood; BI: Bayesian inference; gCF: gene concordance factor; sCF: site concordance factor.

To address these challenges, we developed an extensive statistical pipeline to study the evolutionary patterns in the mitogenomes of Carcharhiniformes (groundsharks), with a particular focus on the contentious Triakidae family (houndsharks; Linck 1790 [30]) [31,32]. Our overarching goal was to evaluate biological signatures in the dataset influencing phylogenetic resolution before selecting the optimal phylogenetic workflow as illustrated in Figure 1. A representative collection of carcharhiniform mitogenomes and outgroups were selected and aligned as described in Section A, whereafter substitution saturation tests were conducted to inform the selection of partitioning schemes as described in Section B. Section C details the mitophylogenomic pipeline used to conduct model selection for ML and Bayesian Inference (BI) analyses and investigate topological conflict around branches of the species tree using concordance factors. Next, the effects of gene-tree conflict on species-tree inference were estimated under the multispecies coalescent model (MSCM) as described in Section D. Lastly, site-heterogeneous models were tested as described in Section E. This protocol can be used to design mitophylogenomic bioinformatic pipelines and serves as educational material for various higher-education modules in molecular evolution.

## Software and datasets

Most of the software programs listed below can be used on Windows 7/8/10/11, Mac OS 10.11 (current versions), and Linux (Ubuntu Desktop LTS, last two supported versions). IQ-Tree has no GUI and must be run through the command line. If you do not have Linux on your device, you can use MobaXTerm v.24.4 (https://mobaxterm.mobatek.net/download.html, last accessed 1/12/2025) for Windows or Tabby Terminal v.1.0.216 (https://tabby.sh/, last accessed 1/12/2025) for Mac to run command line code.

We used a machine with a multi-core central processing unit (CPU) allowing for parallel processing to speed up some of our analyses. The amount of RAM depends on the size of the dataset, but a minimum of 8 GB is recommended. PhyloBayes is primarily developed and distributed for Linux environments. PHYLOBAYES_MPI, as utilised in this protocol, was run through a multicore high-performance computer. The program is designed to take advantage of multiple processors for parallel computing. A multi-core processor or a cluster of machines with MPI (Message Passing Interface) support is recommended for efficient parallelization. The more cores or nodes you can allocate, the faster the analysis may proceed.

If high-performance computing (HPC) resources are not available, the CIPRES (Cyberinfrastructure for Phylogenetic Research) Science Gateway portal v.3.3 at the San Diego Supercomputer Centre [33] (https://www.phylo.org/, last accessed 1/12/2025) is an online platform that provides a user-friendly web interface for performing computationally intensive phylogenetic analyses. MrBayes, PhyloBayes, MAFFT, IQ-Tree, and more can be run through CIPRES. There is also a Geneious plugin. Users can subscribe to a three-month free trial with 1,000 CPU hours; thereafter, CPU hours can be purchased.

**A. Creating alignment datasets**

1. Batch Entrez webserver (https://www.ncbi.nlm.nih.gov/sites/batchentrez, last accessed 1/12/2025) for retrieving GenBank sequences from NCBI

2. GBSEQEXTRACTOR v.0.04 [34] (https://github.com/linzhi2013/gbseqextractor, last accessed 1/12/2025) for extracting genes from GenBank files for alignment. It is run through Biopython [35] (http://www.biopython.org/, last accessed 1/12/2025), which must be pre-installed on your system as per the instructions on the Biopython website. Thereafter, GBSEQEXTRACTOR can be downloaded and saved in the folder where you want to run your analyses

3. MACSE v.2.07 [36] (https://www.agap-ge2pop.org/macsee-pipelines/, last accessed 1/12/2025) for alignment of protein-coding genes. Requires a suitable Java runtime environment

4. MAFFT v.7.299 [37,38] webserver (https://mafft.cbrc.jp/alignment/server/, last accessed 1/12/2025) for alignment of ribosomal RNAs

5. Geneious Prime v.2023.2 [39] (https://www.geneious.com/download/, last accessed 1/12/2025) for alignment editing and comparison. Note that a paid license is required to edit alignments. Mega11 [40] (https://www.megasoftware.net/) is an alternative software that can be used for sequence visualisation and editing. Geneious also provides licenses for students doing courses (see https://www.geneious.com/free-course-license, last accessed 1/12/2025)

6. BMGE v.1.12_1 [41] can be accessed through the NGPhylogeny.fr webserver (https://ngphylogeny.fr/tools/tool/273/form, last accessed 1/12/2025) for cleaning of alignments

7. GenBank files and accession number lists for our dataset are in the folder *2_Galeomorphii_mitogenome_sequences: Data_13_Winn2023_Galeomorphii_mitogenome_seqs*, and concatenated datasets can be found in the folder *3_Multiple_sequence_alignments: Data 14–23* on our Dryad Digital Repository (doi: 10.5061/dryad.sj3tx969h, last accessed 1/12/2025) and GitHub (https://github.com/JessWinn/Houndshark-Mitogenomics, last accessed 1/12/2025)

**B. Testing for substitution saturation and partitioning the data**

1. DAMBE version 7.0.35 [42,43] (http://dambe.bio.uottawa.ca/DAMBE/dambe.aspx, last accessed 1/12/2025) for testing for substitution saturation at different codon positions in different mitogenome regions

2. Multiple sequence alignment datasets for nucleotide and amino acids in fasta format can be found in the folder *3_Multiple_sequence_alignments: Data_14_Galeomorphii_13PCGs_NT.fasta, Data_17_Galeomorphii_13PCGs_2rRNAs_NT.fasta, Data_20_Galeomorphii_13PCGs_AA.fasta* on the Dryad Digital Repository (doi: 10.5061/dryad.sj3tx969h) and GitHub (https://github.com/JessWinn/Houndshark-Mitogenomics)

**C. Mitophylogenomic reconstruction**

1. ModelFinder v.1.6.12 [44] is available through IQ-Tree v.2.1.3 [45] (http://www.iqtree.org/, last accessed 1/12/2025) for data partitioning and evolutionary model selection and maximum likelihood tree construction (with UFBoot2, SH-aLRT, and CF values)

2. Cyberinfrastructure for Phylogenetic Research (CIPRES) Science Gateway portal v.3.3 at the San Diego Supercomputer Centre [33] (https://www.phylo.org/) to run MrBayes v.3.2.6 [46], for Bayesian inference analyses. You may also select to run the program using a multicore high-performance computer if you have access to one

3. R (https://cran.r-project.org/, last accessed 1/12/2025) and R Studio (https://posit.co/products/open-source/rstudio/) for comparing ML tree support values and constructing BI Tracer plots

4. FigTree v.1.4.4 [47] (https://github.com/rambaut/figtree/releases, last accessed 1/12/2025) for tree visualisation. Requires a Java runtime environment

5. Evolview v3 [48] webpage (https://www.evolgenius.info/evolview/, last accessed 1/12/2025) for tree annotation

6. Multiple sequence alignment nucleotide and amino acid datasets in fasta and nexus format can be found in the folder *3_Multiple_sequence_alignments: Data_14_Galeomorphii_13PCGs_NT.fasta, Data_16_Galeomorphii_13PCGs_NT.nex, Data_17_Galeomorphii_13PCGs_2rRNAs_NT.fasta, Data_19_Galeomorphii_13PCGs_2rRNAs_NT.nex, Data_20_Galeomorphii_13PCGs_AA.fasta, and Data_22_Galeomorphii_13PCGs_AA.nex;* partition files in nexus format can be found in the folder *4_Partition_files* on the Dryad Digital Repository and GitHub

**D. Multispecies coalescent modelling**

1. IQ-Tree v.2.1.3 [45] (http://www.iqtree.org/, last accessed 1/12/2025) for gene tree construction

2. ASTRAL v.5.6.3 [49] (https://github.com/smirarab/ASTRAL, last accessed 1/12/2025), a summary-based method to estimate the effects of gene-tree conflict on species-tree inference under the multispecies coalescent model. Java 1.6 or later is required

3. Newick Utilities (https://github.com/tjunier/newick_utils, last accessed 1/12/2025) for collapsing branches with low support for input into ASTRAL. See https://github.com/tjunier/newick_utils/blob/master/doc/nwutils_tutorial.pdf (last accessed 1/12/2025) for specific compiler requirements

4. SVDQuartets [50] in PAUP* v4.0a 169 [51] (https://phylosolutions.com/paup-test/, last accessed 1/12/2025), a site-based method to estimate the effects of gene-tree conflict on species-tree inference under the multispecies coalescent model

5. FigTree v.1.4.4 [47] (https://github.com/rambaut/figtree/releases, last accessed 1/12/2025) for tree visualisation. Requires a Java runtime environment

6. Cleaned and edited gene alignments necessary for ASTRAL were generated in Section C and saved in the folder 2c_CleanEdit. A sample nexus file with gene partitions for our dataset needed to run SVDQuartets can be found in the folder 3_Multiple_sequence_alignments: Data_23_13PCGs_2rRNAs_NT_svd_partitions on the Dryad Digital Repository and GitHub

**E. Testing site heterogenous models**

1. AliGROOVE v.1.08 [2] (https://github.com/PatrickKueck/AliGROOVE, last accessed 1/12/2025) for compositional heterogeneity among lineages and across sites. AliGROOVE is implemented in Perl and uses the Phylo module of the BioPerl library, which is delivered within the package. AliGROOVE GUI is based on C++ and the Qt library

2. BaCoCa v.1.1 [52] (https://github.com/PatrickKueck/BaCoCa, last accessed 1/12/2025) for compositional heterogeneity among lineages and across sites. To execute BaCoCa, a PERL interpreter must be installed. Linux and Mac systems normally contain this as a standard tool, but the additional PERL Statistics::R package must be installed to use the *-r* option of BaCoCa.vX.X.r.pl, which allows the generation of result heat maps by using R. Windows users have to install a PERL interpreter ex post. The developers recommend ActivePerl (http://activeperl.softonic.de/, last accessed 1/12/2025). The additional PERL package Statistics::R can be installed via the ActivePerl package manager and R can be installed from: http://cran.r-project.org/bin/windows/base/ (last accessed 1/12/2025). See https://github.com/PatrickKueck/BaCoCa/blob/master/BaCoCa_Manual.pdf (last accessed 1/12/2025) for more detailed instructions

3. PHYLOBAYES_MPI v.1.9 package [53] (https://github.com/bayesiancook/pbmpi, last accessed 1/12/2025) for Bayesian inference analyses using the pb_mpi program and various site-heterogeneous models.

4. Tracer v.1.7.1 [54] (https://github.com/beast-dev/tracer/releases/tag/v1.7.1, last accessed 1/12/2025) for visualisation and diagnostics of Markov chain Monte Carlo (MCMC) output requiring Java v.1.6 or greater

5. FigTree v.1.4.4 [47] (https://github.com/rambaut/figtree/releases, last accessed 1/12/2025) for tree visualisation. Requires a Java runtime environment

6. The three alignment datasets in fasta and phylip format are found in the folder *3_Multiple_sequence_alignments: Data_14_Galeomorphii_13PCGs_NT.fasta, Data_15_Galeomorphii_13PCGs_NT.phy, Data_17_Galeomorphii_13PCGs_2rRNAs_NT.fasta, Data_18_Galeomorphii_13PCGs_2rRNAs_NT.phy, Data_20_Galeomorphii_13PCGs_AA.fasta, Data_21_Galeomorphii_13PCGs_AA.phy* and the partition file for BaCoCa is found in the folder *4_Partition_files: 13PCGs_2rRNAs_NT.part.txt* on the Dryad Digital Repository and GitHub

## Procedure

**A. Creating alignment datasets**

Here, we provide a streamlined protocol to create alignment datasets for phylogenetic comparison of a collection of mitogenomes. Extracting, aligning, and cleaning gene alignments can be a tedious task, so our goal was to design standardised scripts to expedite the process. The first step is to carefully curate the dataset to contain representative ingroup taxa and suitable outgroups. We describe the process used to curate our Galeomorphii (all modern sharks except the dogfish and its relatives) dataset, which we selected by analysing previous studies conducted on this group. Once the dataset has been obtained, individual genes need to be extracted and aligned to each other separately. Aligning genes separately, rather than aligning full mitogenomes to each other, makes it easier to clean alignments if the length of certain genes varies amongst individuals or when there are alignment gaps. It also makes it easier to check that the reading frame of each protein-coding gene is correct and to remove stop codons. We use *Galeomorphii* and *winn_2023* as identifiers for input and output files in the protocol. Replace these with your own identifiers. If you do not have Linux on your device, you can use MobaXTerm v.24.4 for Windows or Tabby Terminal v.1.0.216 for Mac to run the command line code.

1. Retrieve ingroup and outgroup mitogenomes from GenBank.

a. Copy the GenBank (full) format files for your newly assembled mitogenomes into the folder *1a_Data*.

b. Compile a list of mitogenome accession numbers containing representative mitogenomes for the species, genus, family, order, or superorder you are studying. The mitogenome accession number list and the outgroups can be saved as a genbank.list file.

**Figure 2.**
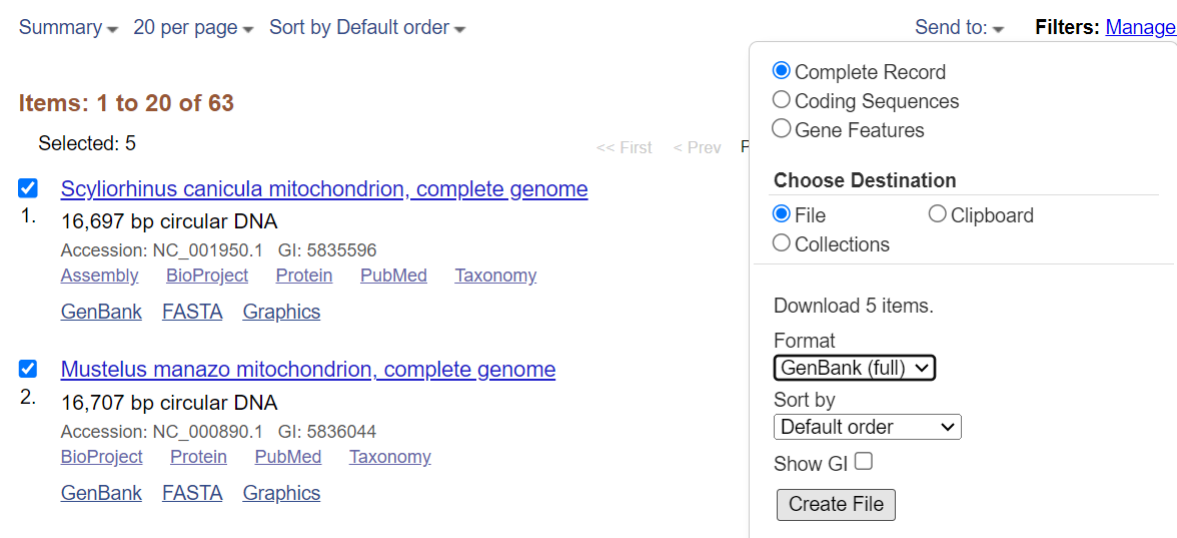
Saving a list of mitogenomes from NCBI ( https://www.ncbi.nlm.nih.gov/ , last accessed 1/12/2025) [55]. Check all the items that appear and make sure they are complete mitogenomes; then, select records and select *Complete Record* and GenBank (full) format before creating the file.

**[Tip 1]** The selection of mitogenomes for your study depends on your research question, previous findings on your study group, and the scope of your study. In our case, we were investigating the mitophylogenomics of the order Carcharhiniformes to better understand the relationships of members designated to the family Triakidae with each other as well as with other families in the order. We obtained our mitogenome list from recent publications by Wang et al. [31] and Kousteni et al. [32] and elected to include four outgroups each from the Lamniformes and Orectolobiformes sister orders.

c. Use the genbank.list file in a Batch Entrez (https://www.ncbi.nlm.nih.gov/sites/batchentrez) search to retrieve the mitogenome records from the *Nucleotide* database.

d. Select all the mitogenome records from NCBI and save them as complete records in GenBank (full) format as a single file (Figure 2). Before saving, make sure all the items selected are complete mitogenome sequences.

e. Merge the GenBank file above and the five newly assembled mitogenomes into one GenBank file.

cat *.gb > winn_2023.gb.

2. Download GBSEQEXTRACTOR as described in the Software and datasets section and save the application, along with *winn_2023.gb*, in a new folder titled *1b_GeneExtract*.

a. Extract rRNA and coding domain sequences (CDS) from the GenBank file into separate fasta files for each rRNA and CDS using the following command line code in a Terminal interface.

# Navigate to the correct working directory

cd ./1b_GeneExtract

# Extracting the rRNAs

gbseqextractor -f winn_2023.gb -prefix winn_2023 -types rRNA -s # output file ´winn_2023.rrna.fasta´

# Extracting the CDS

gbseqextractor -f winn_2023.gb -prefix winn_2023 -types CDS -s # output file `winn_2023.cds.fasta´

b. Merge the rRNA and CDS fasta files.

cat winn_2023.rrna.fasta winn_2023.cds.fasta > winn_2023.cds-rrna.fasta

c. Using Notes, or an equivalent text editor, edit the file *winn_2023.cds-rrna.fasta* to standardise gene names. For instance, some GenBank records denote 12S rRNA as s-rRNA, 12S ribosomal RNA or rrnS, CO1 as COX1, ND2 as nad2, etc. Standardise all genes to the following code: ATP6, ATP8, COX1, COX2, COX3, CYTB, ND1, ND2, ND3, ND4, ND4L, ND5, ND6, 12SrRNA, and 16SrRNA. Save the edited file as *winn_2023.cds-rrna.std.fasta*.

d. Extract individual gene sequences (.fa) from *winn_2023.cds-rrna.std.fasta* using the custom script *maduna2022-gene-extractions.sh*.

GENE=('ATP6' 'ATP8' 'COX1' 'COX2' 'COX3' 'CYTB' 'ND1' 'ND2' 'ND3' 'ND4;' 'ND4L' 'ND5' 'ND6' '12SrRNA' '16SrRNA')

for g in "${GENE[@]}"

do

awk '{ if ((NR>1)&&($0~/^>/)) { printf("\n%s", $0); } else if (NR==1) \

{ printf("%s", $0); } else { printf("\t%s", $0); } }' \

winn_2023.cds-rrna.std.fasta | grep -F $g - | tr "\t" "\n" > "${g}".fa

done

mv 'ND4;.fa' ND4.fa

for f in *.fa

do

sed -i 's/;/_/g' $f

done

3. Create multiple sequence alignments

a. Create a new folder called *2a_MACSE2*. Download the latest version of MACSE and save *macse_v2.06.jar* in the present folder.

b. Upload the extracted PCGs from *1b_GeneExtract* into Geneious and check that each one is in the correct orientation. For example, all coding genes are in the 5′ to 3′ direction in the fish mitochondrial genome, except for ND6 that is in the 3′ to 5′ direction. ND6 has to be selected and changed to the reverse complement by clicking *Sequence | Reverse complement… | Reverse complement entire sequence* and saved as *ND6_rev_comp*. Do some background reading to work out the standard for your species of interest. After confirming the orientation of the PCGs, save them as .fa files in *2a_MACSE2*.

c. Align the PCGs using the for-loop script *maduna2022-13pcgs-msa.sh*.

datadir=./2a_MACSE2

for i in $datadir/*.fa

do

java -jar -Xmx600m macse_v2.06.jar -prog alignSequences -seq "$i" -gc_def 2

done

# alignSequences: aligns nucleotide (NT) coding sequencing using their amino acid (AA) translations.

# gc_def: specify the genetic code 2 (The_Vertebrate_Mitochodnrial_Code) or change according to your study taxa.

**Figure 3.**
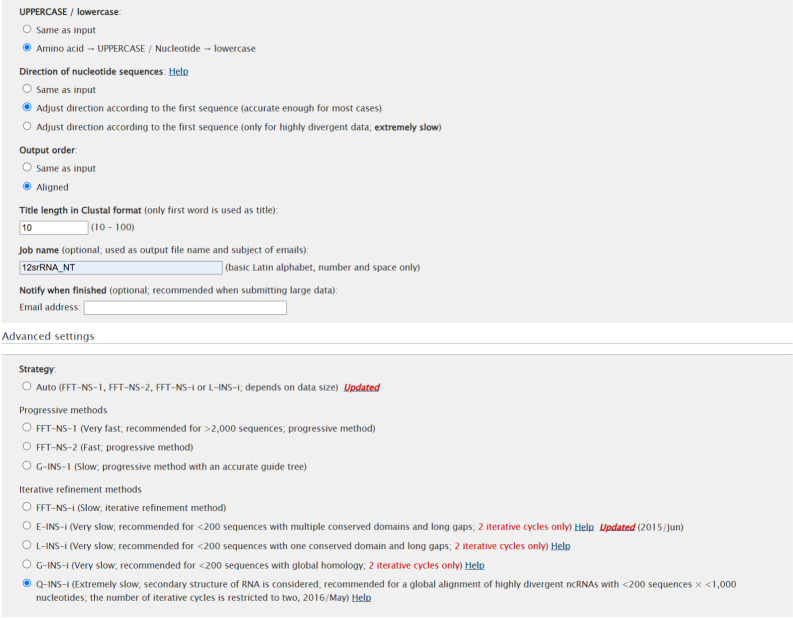
Parameter settings for the alignment of rRNAs on the MAFFT v.7.299 webserver [37,38] ( https://mafft.cbrc.jp/alignment/server/ ). Upload rRNA sequence lists in fasta format. Adjust alignment direction according to the first sequence and select the Q-INS-I iterative refinement method. Leave all other parameters on default settings.

**Figure 4.**
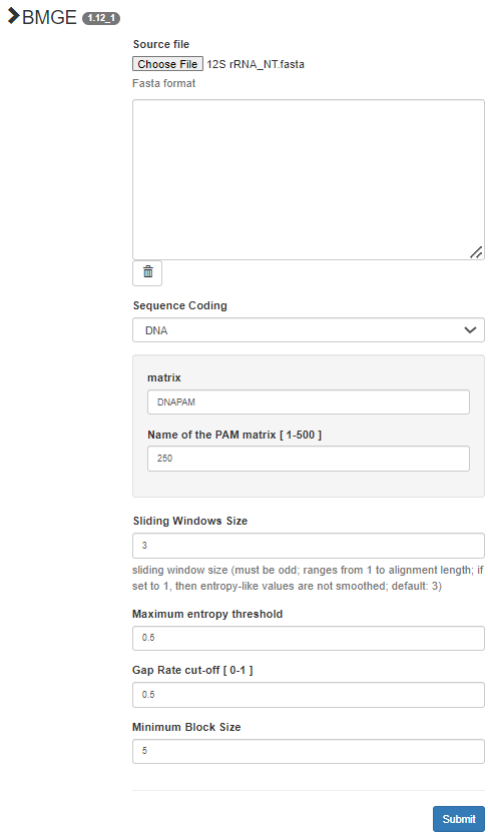
Alignment cleaning in BMGE v.1.12_1 [41] ( https://ngphylogeny.fr/tools/tool/273/form ). Upload the alignment files in fasta format. Select *DNA* for *Sequence Coding* and leave other parameters on default.

**Figure 5.**
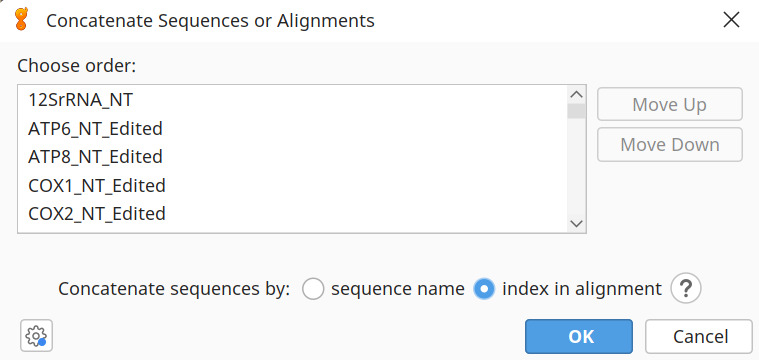
Concatenating the alignments of the 13 protein-coding genes (PCGs) to create Dataset 1: 13PCGS_NT in Geneious v.2024.0.2 [39]. Ensure species names are sorted alphabetically in each alignment and then select to concatenate sequences by the *index in alignment*.

**Figure 6.**
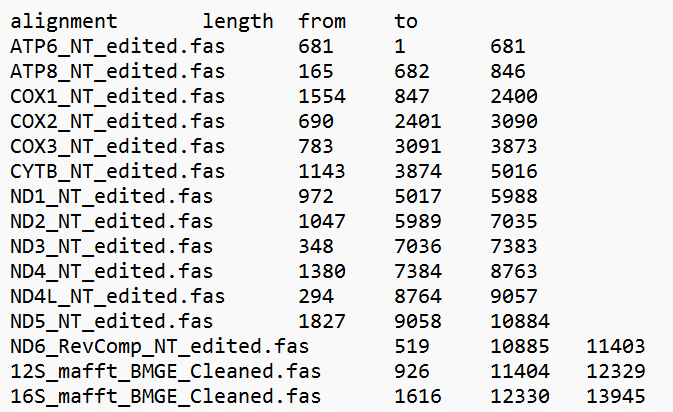
Mitogenome length summary for *13PCGs_2rRNAs_NT* (Dataset 2). This can be saved from the alignment information in Geneious v.2024.0.2 [39].

d. Insert the rRNA genes extracted in step B2a into the online version of MAFFT (https://mafft.cbrc.jp/alignment/server/index.html). Select the Q-INS-i iterative refinement method, adjusting the direction according to the first sequences confirmed to be in the 5′ to 3′ direction and leaving all other parameters on default (see Figure 3 for parameters).

e. Open all alignments from *2a_MACSE* in Geneious, change the translation settings to the species-specific codon used for your dataset [Vertebrate Mitochondrial (transl_table 2) for our dataset], remove stop codons, and ensure each alignment length is divisible by 3. Only codons coding for amino acids should be included in downstream phylogenetic analyses. Start codons are retained because they still code for amino acids. The common start codon, ATG, codes for methionine. Occasionally, other start codons, such as GTG, are used for mitochondrial PCGs.

f. If there are remaining ambiguously aligned sites, remove them with BMGE maintaining default settings (Figure 4). Clean both the rRNA alignments with BMGE. BMGE (Block Mapping and Gathering with Entropy) is a bioinformatics tool used to clean and filter multiple sequence alignments (MSA) using an entropy-based approach to evaluate the variability of columns (alignment positions) in an MSA [41]. High-entropy columns, which often indicate poorly aligned or highly variable regions, are flagged for removal, and conserved regions that are more reliable for downstream phylogenetic and functional analyses are retained. It can exclude gaps, poorly aligned positions, and noisy regions to produce a cleaner alignment. Alignment cleaning is not necessary when aligning very similar mitogenomes (for example, individuals belonging to the same species) but can be useful when aligning mitogenome regions from different species, which often vary in length.

g. Export the edited alignments into the folder *2c_CleanEdit*.

h. Import the cleaned and edited alignments back into Geneious. Open each alignment in alignment view and then right-click on the identity heading. Click *Sort | By Name*.

i. Now, select all 13 of the PCG alignments and click *Tools | Concatenate Sequences or Alignments | Concatenate* (Figure 5). Save as *Galeomorphii_13PCGs_NT* (Dataset 1) in fasta, nexus, and phylip format (these files are listed as Data 14–16 on the Dryad Repository) in *2d_ConCat*.

j. Concatenate the 13 PCGs and 2 rRNA genes in Geneious and save as *Galeomorphii_13PCGs_2rRNAs_NT* (Dataset 2) in fasta, nexus, and phylip format (Data 17–19).

k. Translate *Galeomorphii_13PCGs_NT* and save as *Galeomorphii_13PCGs_AA* (Dataset 3) in fasta, nexus, and phylip format (Data 20–22).

l. Make and save length summaries with the length and alignment locations of each alignment from the alignment information to use for the partition files (an example for Dataset 2 is shown in Figure 6).

**B. Testing for substitution saturation and partitioning the data**

This protocol describes an approach for selecting a priori partitioning schemes for a dataset to use in phylogenetic reconstruction. The first step is to evaluate substitution saturation at each codon position of the 13PCGs as well as the entire *13PCGs_NT* and *13PCGs_2rRNAs_NT* datasets in DAMBE. Second, substitution saturation is visually inspected by plotting the number of transitions (s) vs. transversions (v) vs. divergence. These results are then evaluated to select a priori partitioning strategies to test during phylogenetic reconstruction. Substitution saturation occurs when multiple substitutions have occurred at the same site, compromising the signal in the dataset, and making it difficult to accurately infer the true historical relationships between sequences. A gene or codon position that is saturated could be omitted to improve the reliability of phylogenetic analyses. For example, if codon position 3 possesses a high degree of substitution saturation across the PCGs and the entire alignment dataset, it can be excluded from a priori partitioning schemes. The a priori schemes presented here were selected based on the evolutionary signatures of our dataset, which showed little substitution saturation. It may be necessary to consider alternatives for your own dataset.

See the following chapter from “The Phylogenetic Handbook” for details on assessing substitution saturation with DAMBE: http://dambe.bio.uottawa.ca/publications/2009PhylHandbookChap20.pdf (last accessed 1/12/2025).

**Figure 7.**
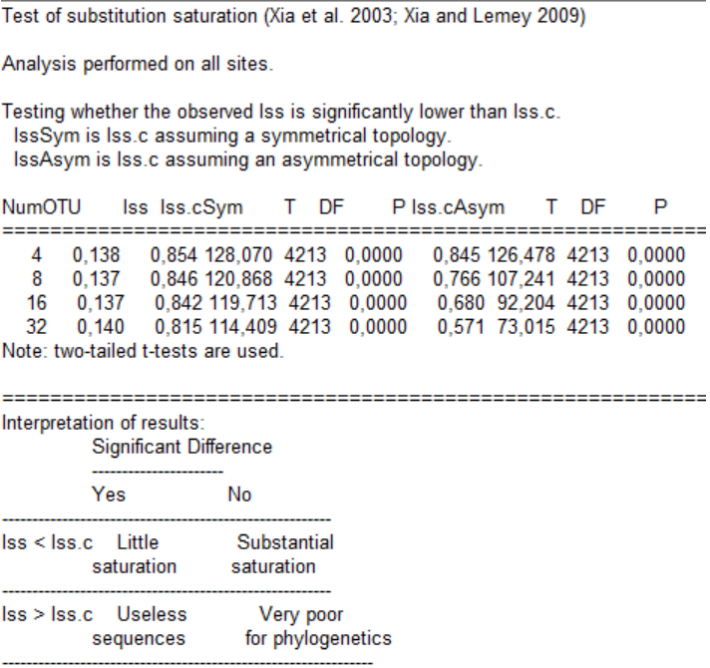
Output for two-tailed tests to examine the degree of nucleotide substitution saturation for codon position 1 and 2 of the 13PCG_NT (Dataset 1), considering the proportion of invariant sites in DAMBE [42,43]. NumOTU: number of taxonomic units; Iss: index of substitution saturation; Iss.cSym: critical Iss assuming symmetrical topology; T: t-value; P: probability Iss < Iss.cSym; Iss.cAsym: critical Iss assuming asymmetrical topology; P: probability Iss < Iss.c Asym.

1. Perform two-tailed tests to examine the degree of nucleotide substitution saturation [56] for each codon position of the 13PCGs, the PCGs as a whole, and the entire 13_PCGs_NT and 13PCGs_rRNAs_NT datasets, taking into account the proportion of invariant sites as recommended by Xia and Lemey [57] in DAMBE.

a. Copy the cleaned and edited gene alignments in fasta format from Section A saved in *2c_CleanEdit* as well as the concatenated alignments from *2d_ConCat* into the folder *3_DAMBE*.

b. Open DAMBE and click *File | Open standard sequence file*. A window will appear with input options. Select *Protein-coding Nuc. Seq.*, choose the relevant genetic code for PCGs (Table 2: Vertebrate mitochondrial for our dataset), and select *Non-Protein Nuc. Seq.* for rRNAs.

c. Begin by selecting the portion of the alignment you want to work on. To assess substitution saturation at codon positions 1 and 2 of the 13PCGs, click *Sequences | Work on codon positions 1 and 2*. We investigated substitution saturation at codon positions 1, 3, 1 + 2 combined, and all positions.

d. Estimate the proportion of invariant sites [P(inv)] by clicking *Seq. Analysis | Substitution rates over sites | Estimate proportion of invariant sites*. Specify *Use a new tree* and a window will appear providing a choice of tree-building algorithms and options. Choose the *Neighbour-Joining* algorithm, keep the default settings, click *Run*, and then *Go!* At the end of the text output, the estimated P(inv) is shown as P(invariate) = 0,18763.

e. Now, go to *Seq. Analysis | Measure Substitution Saturation | Test by Xia et al.*, input 0,18763 as the P(inv), and click *Go!* The following output is generated (Figure 7).

**[Tip 2]** From the output, we are interested in comparing the index of substitution saturation (Iss) to the critical Iss (the threshold value) assuming a symmetrical topology and then assuming an asymmetrical topology. The critical Iss is a measure of the extent to which substitutions in your sequence data have reached saturation. If the Iss is below the critical value (p < 0.05: Iss < Iss.c), the data is not saturated to a problematic extent, and phylogenetic inferences are likely to be reliable. If the Iss exceeds the critical value (p > 0.05: Iss > Iss.c), it indicates that saturation is potentially compromising the phylogenetic signal in the data. Symmetrical and asymmetrical topologies refer to the arrangement of branches in a phylogenetic tree. Symmetrical topologies have a balanced, tree-like structure, while asymmetrical topologies may have imbalances or deviations from the expected tree shape. The assessment of substitution saturation may vary between symmetrical and asymmetrical topologies, as saturation can impact various parts of the tree differently, which is why we assessed both. See Table 1 for how we configured our results table from the output values, using 32 OTUs considering that our dataset contains 64 sequences.

**Table 1.** Indices from two-tailed tests of substitution saturation accounting for the proportion of invariant sites for each protein-coding gene, two rRNAs, and the full 13PCGs_NT and 13PCGs_2rRNAs datasets. P(inv): proportion of invariant sites; Iss: index of substitution saturation; Iss.c Sym: critical Iss assuming symmetrical topology; Ps: probability Iss < Iss.c Sym; Iss.c Asym: critical Iss assuming asymmetrical topology; Pa: probability Iss < Iss.c Asym. Results were generated using DAMBE [42,43].

Partition	P(inv)	Iss	Iss.c Sym	Ps	Iss.c Asym	Pa
atp6	0.168	0.247	0.721	0.000	0.395	0.000
atp6_pos1+2	0.219	0.11	0.697	0.000	0.37	0.000
atp6_pos3	0.013	0.526	0.685	0.000	0.364	0.000
atp8	0.115	0.258	0.707	0.000	0.408	0.000
atp8_pos1+2	0.168	0.146	0.778	0.000	0.539	0.000
atp8_pos3	0.03	0.48	1.099	0.000	1.097	0.000
cox1	0.197	0.201	0.777	0.000	0.495	0.000
cox1_pos1+2	0.308	0.046	0.75	0.000	0.447	0.000
cox1_pos3	0.019	0.494	0.704	0.000	0.378	0.000
cox2	0.189	0.175	0.722	0.000	0.397	0.000
cox2_pos1+2	0.274	0.049	0.698	0.000	0.371	0.000
cox2_pos3	0.189	0.176	0.722	0.000	0.397	0.000
cox3	0.181	0.196	0.73	0.000	0.411	0.000
cox3_pos1+2	0.265	0.07	0.704	0.000	0.378	0.000
cox3_pos3	0.022	0.454	0.682	0.000	0.356	0.001
Cytb	0.146	0.239	0.757	0.000	0.459	0.000
Cytb_pos1+2	0.222	0.103	0.728	0.000	0.408	0.000
Cytb_pos3	0.017	0.527	0.689	0.000	0.36	0.000
nad1	0.167	0.239	0.746	0.000	0.439	0.000
nad1_pos1+2	0.24	0.101	0.717	0.000	0.391	0.000
nad1_pos3	0.021	0.523	0.684	0.000	0.354	0.000
nad2	0.14	0.285	0.751	0.000	0.448	0.000
nad2_pos1+2	0.2	0.154	0.722	0.000	0.398	0.000
nad2_pos3	0.016	0.559	0.686	0.000	0.356	0.000
nad3	0.154	0.225	0.686	0.000	0.356	0.000
nad3_pos1+2	0.191	0.11	0.684	0.000	0.362	0.000
nad3_pos3	0.034	0.443	0.765	0.000	0.516	0.110
nad4	0.143	0.256	0.77	0.000	0.482	0.000
nad4_pos1+2	0.229	0.121	0.742	0.000	0.431	0.000
nad4_pos3	0.012	0.537	0.698	0.000	0.371	0.000
nad4L	0.125	0.25	0.682	0.000	0.354	0.001
nad4L_pos1+2	0.213	0.104	0.692	0.000	0.379	0.000
nad4L_pos3	0.016	0.563	0.811	0.000	0.598	0.470
nad5	0.156	0.255	0.785	0.000	0.512	0.000
nad5_pos1+2	0.211	0.108	0.761	0.000	0.467	0.000
nad5_pos3	0.012	0.542	0.713	0.000	0.386	0.000
nad6	0.126	0.266	0.704	0.000	0.378	0.000
nad6_pos1+2	0.203	0.138	0.686	0.000	0.356	0.000
nad6_pos3	0.01	0.533	0.702	0.000	0.399	0.000
12S	0.182	0.154	0.742	0.000	0.432	0.000
16S	0.428	0.211	0.779	0.000	0.5	0.000
13PCGs	0.181	0.244	0.818	0.000	0.572	0.000
13PCGs_pos1+2	0.446	0.138	0.815	0.000	0.571	0.000
13PCGs_pos3	0.022	0.502	0.809	0.000	0.555	0.000
13PCGs_2rRNAs	0.188	0.227	0.819	0.000	0.573	0.000

**Figure 8.**
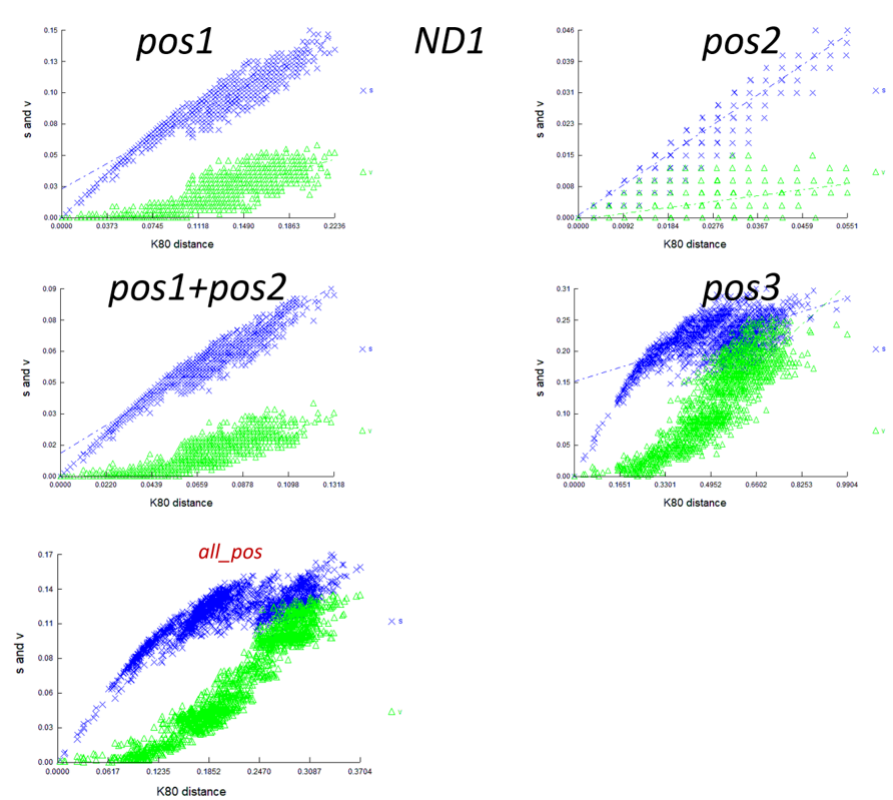
Plot of transitions (s) and transversions (v) vs. divergence based on genetic distances derived from the Kimura two-parameter (K80 distance) for the *ND1* gene generated using DAMBE v.7.0.35 [42,43]. Plots were constructed for codon position one (pos1), two (pos2), one and two excluding three (pos1 + pos2), three (pos3) and all positions together (all_pos).

2. Visually inspect substitution saturation by plotting the number of transitions (s) and transversions (v) vs. divergence for codon positions one, two, three, one and two, and all positions for each gene as well as the concatenated datasets in DAMBE (Figure 8).

a. Click *Seq. Analysis | Nucleotide substitution pattern | Detailed Output*. Add all sequence pairs to the right window and click *Run*.

b. A window will pop up asking if you want to plot the number of transitions and transversions vs. Kimura’s two-parameter distance. Click *Yes*.

**[Tip 3]** This plot is based on genetic distances derived from the Kimura two-parameter (K2P or K80) substitution model [58]. The K80 model assumes that transitions and transversions occur at different rates and accounts for unequal base frequencies. In the absence of strong biases or evolutionary forces, you expect to see a distribution of points around the diagonal because, over time, transitions and transversions accumulate in a roughly equal manner. A noticeable trend away from the diagonal may suggest a bias in the mutation process. If the plot shows more transitions than transversions (or vice versa), it indicates a specific bias in the types of mutations occurring. If the plot reaches a plateau, this could indicate saturation, where the sequences have accumulated multiple mutations, and additional divergence does not result in a significant increase in substitutions. We constructed plots for all codon positions of the PCGs and rRNAs and for the *13PCGs_NT* and *13PCGs_2rRNAs_NT* combined.

3. Construct partition nexus files for each dataset based on the substitution saturation results.

a. Dataset 1 & 3: one partition for the entire alignment, 13 partitions for each PCG.

b. Dataset 2: one partition for the entire alignment, five partitions (codon: pos1 + pos2 + pos3 + 2 rRNAs), four partitions (codon: pos1_pos2 + pos3 + 2 rRNAs), four partitions (codon: pos1 + pos3 + 2 rRNAs), 15 partitions for each PCG and 2 rRNAs, 41 partitions (gene × codon: 13 PCGs pos1 + 13 pos2 + 13 pos2 + 2 rRNAs), 28 partitions (gene × codon: 13 PCGs pos1_pos2 + 13 PCGs pos3 + 2 rRNAs), 28 partitions (gene × codon: 13 PCGs pos1 + 13 PCGs pos3 + 2 rRNAs).

**[Tip 4]** We partitioned our datasets based on codons (PS02–PS04), genes (PS05/PS05AA), and genes × codons (PS06–PS08) and tested the dataset without partitions (PS01/PS01AA) as a control. Our substitution saturation results show that there is minimal saturation across sites and genes, and codon position 2 does not improve the informativity of the analysis; so, we elected to test scenarios where the datasets were partitioned at all codon positions, combining position 1 and 2 and omitting position 2 for the codon and gene × codon strategies. Table 2 shows the different partitioning strategies used for our three datasets. You may elect to test different partitioning strategies based on your substitution saturation results or exclude genes that contain significant substitution saturation, which may negatively affect the resolution of your phylogenetic trees.

**Table 2.** The a priori partitioning schemes tested in this study. PCG: protein-coding gene; pos.: position; AA: amino acid.

Partition scheme	Partitions	Number of partitions
PS01	None (1 partition)	1
PS01AA	None (1 partition)	1
PS02	Codon: pos1 + pos2 + pos3 + 2 rRNAs	5
PS03	Codon: pos1_pos2 + pos3 + 2 rRNAs	4
PS04	Codon: pos1 + pos3 + 2 rRNAs	4
PS05	Gene: 13 PCGs + 2 rRNAs	15
PS05AA	Gene: 13 PCGs	13
PS06	Gene × codon: 13 PCGs pos1 + 13 pos2 + 13 pos2 + 2 rRNAs	41
PS07	Gene × codon: 13 PCGs pos1_pos2 + 13 PCGs pos3 + 2 rRNAs	28
PS08	Gene × codon: 13 PCGs pos1 + 13 PCGs pos3 + 2 rRNAs	28

**C. Mitophylogenomic reconstruction**

Here, we describe how to conduct phylogenetic reconstruction using a partitioned super-matrix with different evolutionary models applied to the partitions. First, each of the predefined a priori partitioning schemes selected in Section B are fed into ModelFinder v.1.6.12 [21] in IQ-Tree v.2.1.3 [45], and the best-fitting evolutionary models and partitions are selected for each. Next, phylogenetic reconstruction under a maximum likelihood (ML) framework is conducted using these evolutionary models and partitions. Lastly, an approximately unbiased (AU) tree topology test [59] can be performed to determine how the a priori partitioning strategy influences the phylogenetic outcome and guide the selection of the most appropriate partitioning scheme to use for the dataset. We also discuss the process of conducting secondary model selection in IQ-Tree to select the next best model to apply during Bayesian inference (BI) with MrBayes v.3.2.6 [46].

Additionally, we demonstrate how to assess topological conflict around each branch of the species tree by calculating gene (gCF) and site concordance factors (sCF) in IQ-Tree. The gCF and sCF for each branch of the species tree indicate the percentage of gene trees and alignment sites, respectively, that support that branch [59]. The sCF values have a minimum frequency of approximately 30%, as they compare the three possible quartet resolutions around a node. When data provide no clear preference among these resolutions, the expected sCF is approximately 33%. In contrast, gCF values can be as low as 0% if no gene tree includes a branch present in the species tree. This happens because gCF is calculated from full gene trees, which involve many more possible resolutions than just three [59]. A combination of biological factors and stochastic errors can lead to such gene-tree discordance. To determine whether ILS is the cause of such discordance, a χ2-test is conducted using Lanfear’s R script [59] to decide whether the frequency of gene trees (gCF) and sites (sCF) supporting the two alternative topologies differ significantly.

We also explain how to compare gCF and sCF values with UFBoot2 values from the ML analyses using Lanfear’s R script. Such comparisons help provide insight into the robustness and reliability of phylogenetic inferences conducted for the dataset. The script is written for our Galeomorphii sample files. Insert your own designations to run the scripts for your own dataset.

See the IQ-Tree manual for detailed instructions on how to select parameters and run the program as well as output file descriptions: http://www.iqtree.org/doc/. Additionally, this tutorial (https://www.robertlanfear.com/blog/files/concordance_factors.html) by Robert Lanfear provides an in-depth explanation of interpreting concordance factor values and conducting χ2-tests to compare concordance factors and bootstrap values. Lastly, see https://pmc.ncbi.nlm.nih.gov/articles/PMC5624502/ (last accessed 1/12/2025) for guidance on Bayesian phylogenetic analyses [61].

1. Use ModelFinder in IQ-Tree to determine the best partitioning scheme and corresponding evolutionary models to use in an ML analysis using the corrected Akaike Information Criterion (AICc) and the edge-linked proportional partition model [62].

a. Create a folder titled *4a_ML*. In this directory, create folders titled *13PCGs_2rRNAs* and *13PCGs* containing the concatenated multiple sequence alignments in fasta format (Data 14, 17, and 20 of the sample files) and then create subfolders *PS01 – PS08*. Place the partition files created in step B3 into their relevant subfolder.

b. Run the following script on the command line for each dataset:

*# Dataset 1: 13PCGs_NT*

iqtree2 -s Data_14_Galeomorphii_13PCGs_NT.fasta -st DNA -p PS01/PS01.nex -pre PS01/PS01_run01_mf -m MF+MERGE -AICc -rcluster 30 -T 3 -cmax 20

iqtree2 -s Data_14_Galeomorphii_13PCGs_NT.fasta -st DNA -p PS05/PS05.nex -pre PS05/PS05_run01_mf -m MF+MERGE -AICc -rcluster 30 -T 3 -cmax 20

*# Dataset 2: 13PCGs_2rRNAs_NT*

iqtree2 -s Data_17_Galeomorphii_13PCGs_2rRNAs_NT.fasta -st DNA -p PS01/PS01.nex -pre PS01/PS01_run01_mf -m MF+MERGE -AICc -rcluster 30 -T 3 -cmax 20

iqtree2 -s Data_17_Galeomorphii_13PCGs_2rRNAs_NT.fasta -st DNA -p PS02/PS02.nex -pre PS02/PS02_run01_mf -m MF+MERGE -AICc -rcluster 30 -T 3 -cmax 20

iqtree2 -s Data_17_Galeomorphii_13PCGs_2rRNAs_NT.fasta -st DNA -p PS03/PS03.nex -pre PS03/PS03_run01_mf -m MF+MERGE -AICc -rcluster 30 -T 3 -cmax 20

iqtree2 -s Data_17_Galeomorphii_13PCGs_2rRNAs_NT.fasta -st DNA -p PS04/PS04.nex -pre PS04/PS04_run01_mf -m MF+MERGE -AICc -rcluster 30 -T 3 -cmax 20

iqtree2 -s Data_17_Galeomorphii_13PCGs_2rRNAs_NT.fasta -st DNA -p PS05/PS05.nex -pre PS05/PS05_run01_mf -m MF+MERGE -AICc -rcluster 30 -T 3 -cmax 20

iqtree2 -s Data_17_Galeomorphii_13PCGs_2rRNAs_NT.fasta -st DNA -p PS06/PS06.nex -pre PS06/PS06_run01_mf -m MF+MERGE -AICc -rcluster 30 -T 3 -cmax 20

iqtree2 -s Data_17_Galeomorphii_13PCGs_2rRNAs_NT.fasta -st DNA -p PS07/PS07.nex -pre PS07/PS07_run01_mf -m MF+MERGE -AICc -rcluster 30 -T 3 -cmax 20

iqtree2 -s Data_17_Galeomorphii_13PCGs_2rRNAs_NT.fasta -st DNA -p PS08/PS08.nex -pre PS08/PS08_run01_mf -m MF+MERGE -AICc -rcluster 30 -T 3 -cmax 20

*# Dataset 3: 13PCGs_AA*

iqtree2 -s Data_20_Galeomorphii_13PCGs_AA.fasta -st AA -p PS01/PS01AA.nex -pre PS01/PS01AA_run01_mf -m MF+MERGE -AICc -rcluster 30 -T 3 -cmax 20

iqtree2 -s Data_20_Galeomorphii_13PCGs_AA.fasta -st AA -p PS05/PS05AA.nex -pre PS05/PS05AA_run01_mf -m MF+MERGE -AICc -rcluster 30 -T 3 -cmax 20

**[Tip 5]** We applied the new model selection procedure *(-m MF+MERGE*), which additionally implements the FreeRate heterogeneity model, inferring the site rates directly from the data instead of being drawn from a gamma distribution (-cmax 20; [63]). We set the maximum number of rate categories, cmax, to 20 since it is likely that more rate variations will be observed for alignments with a greater number of sequences. The top 30% partition schemes were checked using the relaxed clustering algorithm (*−rcluster 30*), as described in Lanfear et al. [15]. Three CPU cores (*-T*) are used to decrease computational burden. *-T 3* was determined to be suitable for our dataset; however, you can use *-T AUTO* in a test run for one partitioning scheme to first determine the number of CPU cores to use for your dataset before running all the analyses. Depending on the computing system, it might be required to set an upper limit of CPU cores that can automatically be assigned using the *-ntmax* option. Most standard computing systems have at least two CPU cores. See the IQ-Tree manual for more details on these parameters (http://www.iqtree.org/doc/) and finetune them to suit your dataset requirements.

c. The analysis produces various output files. More detailed descriptions of these can be found in the IQ-Tree manual. In the next steps, we will describe which files we made use of.

2. Apply secondary model selection for the best-fitting partitions identified by ModelFinder in step C1 under the FreeRate heterogeneity model to select the next best model for Bayesian inference.

a. Use the *(PS01-PS08)_best_model.nex* files as input files and rerun ModelFinder with options: *-m TESTONLY -mset mrbayes* to restrict the results to models supported by MrBayes.

b. Create a directory title *4b_BI* with a subfolder *1_Model_Selection* and copy the same folder format described above for the ML analysis. Copy the corresponding *run01_mf.best_scheme.nex* file created in the first run above into each partition scheme folder.

c. In the command line, execute the following scripts for each dataset:

*# Dataset 1: 13PCGs_NT*

iqtree2 -s Data_14_Galeomorphii_13PCGs_NT.fasta -st DNA -p PS01/PS01_run01_mf.best_scheme.nex -pre PS01/PS01_run01_mf -m TESTONLY -AICc -T AUTO -mset mrbayes

iqtree2 -s Data_14_Galeomorphii_13PCGs_NT.fasta -st DNA -p PS05/PS05_run01_mf.best_scheme.nex -pre PS05/PS05_run01_mf -m TESTONLY -AICc -T AUTO -mset mrbayes

*# Dataset 2: 13PCGs_2rRNAs*

iqtree2 -s Data_17_Galeomorphii_13PCGs_2rRNAs_NT.fasta -st DNA -p PS01/PS01_run01_mf.best_scheme.nex -pre PS01/PS01_run01_mf -m TESTONLY -AICc -T AUTO -mset mrbayes

iqtree2 -s Data_17_Galeomorphii_13PCGs_2rRNAs_NT.fasta -st DNA -p PS02/PS02_run01_mf.best_scheme.nex -pre PS02/PS02_run01_mf -m TESTONLY -AICc -T AUTO -mset mrbayes

iqtree2 -s Data_17_Galeomorphii_13PCGs_2rRNAs_NT.fasta -st DNA -p PS03/PS03_run01_mf.best_scheme.nex -pre PS03/PS03_run01_mf -m TESTONLY -AICc -T AUTO -mset mrbayes

iqtree2 -s Data_17_Galeomorphii_13PCGs_2rRNAs_NT.fasta -st DNA -p PS04/PS04_run01_mf.best_scheme.nex -pre PS04/PS04_run01_mf -m TESTONLY -AICc -T AUTO -mset mrbayes

iqtree2 -s Data_17_Galeomorphii_13PCGs_2rRNAs_NT.fasta -st DNA -p PS05/PS05_run01_mf.best_scheme.nex -pre PS05/PS05_run01_mf -m TESTONLY -AICc -T AUTO -mset mrbayes

iqtree2 -s Data_17_Galeomorphii_13PCGs_2rRNAs_NT.fasta -st DNA -p PS06/PS06_run01_mf.best_scheme.nex -pre PS06/PS06_run01_mf -m TESTONLY -AICc -T AUTO -mset mrbayes

iqtree2 -s Data_17_Galeomorphii_13PCGs_2rRNAs_NT.fasta -st DNA -p PS07/PS07_run01_mf.best_scheme.nex -pre PS07/PS07_run01_mf -m TESTONLY -AICc -T AUTO -mset mrbayes

iqtree2 -s Data_17_Galeomorphii_13PCGs_2rRNAs_NT.fasta -st DNA -p PS08/PS08_run01_mf.best_scheme.nex -pre PS08/PS08_run01_mf -m TESTONLY -AICc -T AUTO -mset mrbayes

*# Dataset 3: 13PCGs_AA*

iqtree2 -s Data_20_Galeomorphii_13PCGs_AA.fasta -st AA -p PS01/PS01AA_run01_mf.best_scheme.nex -pre PS01/PS01AA_run01_mf -m TESTONLY -AICc -T AUTO -mset mrbayes

iqtree2 -s Data_20_Galeomorphii_13PCGs_AA.fasta -st AA -p PS05/PS05AA_run01_mf.best_scheme.nex -pre PS05/PS05AA_run01_mf -m TESTONLY -AICc -T AUTO -mset mrbayes

d. Evaluate the likelihood statistics to select the best partitioning scheme (Table 3). These values can be found in the *run01_mf.iqtree* and *run02_mf.iqtree* files generated by ModelFinder for the ML analyses. The lower the AICc/BIC and higher the log-likelihood scores, the better the partitioning strategy, and corresponding models, fit the dataset.

**Table 3.** Likelihood statistics for the eight a priori partitioning schemes used to search for the best-fit partitioning scheme and models of evolution for maximum likelihood tree construction in ModelFinder with the corrected Akaike information criterion (AICc); edge-linked proportional partition model as implemented in IQ-Tree v.2.2.0.3 [45].

Partition scheme	lnL	NFP	NDB	AICc	TlnL	TlnL(SE)	TlnL(WT)	TNP	TAIC	TAICc	TBIC	TTL	TSIBL	%SIBL_TL
PS01	-208933.40	143		418155.78	-208905.19	2002.15	-79201.67	143	418096.37	418099.35	419175.00	6.07	1.81	29.81
PS02	-202881.48	203	5	406174.99	-202847.04	1975.85	-67943.12	129	405952.07	405954.50	406925.10	6.78	2.13	31.47
PS03	-203624.55	190	4	407634.37	-203596.71	1978.12	-69512.15	128	407449.42	407451.81	408414.91	6.77	2.11	31.25
PS04	-187038.42	190	4	374464.13	-187019.50	1772.40	-57659.58	128	374295.01	374298.30	375219.76	9.14	2.92	31.95
PS05	-206969.08	263	10	414474.31	-206953.65	1979.62	-66839.63	134	414175.30	414177.92	415186.04	6.34	1.92	30.31
PS06	-205561.29	412	24	411969.95	-205540.63	1998.50	-60105.46	148	411377.26	411380.24	412504.20	6.88	2.23	32.46
PS07	-206467.92	339	17	413629.59	-206451.82	2002.08	-61855.61	141	413185.64	413188.34	414259.27	6.82	2.20	32.18
PS08	-189997.55	301	15	380613.81	-189962.61	1825.61	-52598.75	139	380203.21	380206.74	381220.93	9.09	3.00	33.02

lnL: Log-likelihood of the tree.

NFP: Number of free parameters (#branches + #model parameters).

NDB: Number of data blocks.

AICc: Corrected Akaike information criterion.

TlnL: Total log-likelihood of the tree.

TlnL(SE): Total log-likelihood of the tree (standard deviation).

TlnL(WT): Unconstrained log-likelihood (without tree).

TFP: Total number of free parameters (#branches + #model parameters).

TAIC: Total Akaike information criterion.

TAICc: Total corrected Akaike information criterion.

TBIC: Total Bayesian information criterion.

TTL: Total tree length (sum of branch lengths).

TSIBL: Total sum of internal branch lengths.

%SIBL_TL: Percentage of total sum of internal branch length as a percentage of tree length.

3. Compare the substitution models and partitions selected based on AICc for ML and BI analyses (Table 4). This information can be found in the *best_model.nex* files produced in IQ-Tree. Take note of how the a priori partitions you defined in Section B are grouped together for each partition scheme. Are the same substitution models selected for a particular mitogenome region across partition schemes? You will notice that the BI substitution models are simpler than the ML models because not all ML models are available for BI analyses.

**Table 4.** Best-fit partition schemes and substitution models determined using ModelFinder [21] in IQ-Tree [45] for maximum likelihood (ML) and Bayesian inference (BI) phylogenies informed by the corrected Akaike information criterion (AICc).

Partition scheme	Partition (AICc)	Best fit substitution model (AICc)	
		**ML**	**BI**
PS01	All	GTR+F+I+I+R6	GTR+F+I+G4
PS02	13PCGs_pos1	GTR+F+R4	GTR+F+I+G4
	13PCGs_pos2	GTR+F+R3	GTR+F+I+G4
	13PCGs_pos3	GTR+F+R6	GTR+F+I+G4
	12S	GTR+F+R5	GTR+F+I+G4
	16S	GTR+F+R4	GTR+F+I+G4
PS03	13PCGs_pos1_pos2	TIM2+F+R5	GTR+F+I+G4
	13PCGs_pos3	GTR+F+R6	GTR+F+I+G4
	12S	GTR+F+R5	GTR+F+I+G4
	16S	GTR+F+R4	GTR+F+I+G4
PS04	13PCGs_pos1	GTR+F+R4	GTR+F+I+G4
	13PCGs_pos3	GTR+F+R6	GTR+F+I+G4
	12S	GTR+F+R5	GTR+F+I+G4
	16S	GTR+F+R4	GTR+F+I+G4
PS05	ATP6	TIM2+F+R4	GTR+F+I+G4
	ATP8	TPM2+F+I+G4	HKY+F+I+G4
	COX1	GTR+F+R4	GTR+F+I+G4
	COX2	TIM2+F+R4	GTR+F+I+G4
	COX3_ND3	TIM2+F+R5	GTR+F+I+G4
	CYTB_ND1_ND4_ND4L_ND5	TIM2+F+R6	GTR+F+I+G4
	ND2	TIM2+F+R5	GTR+F+I+G4
	ND6	GTR+F+I+G4	GTR+F+I+G4
	12S	GTR+F+R5	GTR+F+I+G4
	16S	GTR+F+R4	GTR+F+I+G4
PS06	ATP6_pos1_ND1_pos1_ND3_pos1_ND4L_pos1	TIM2+F+R4	GTR+F+I+G4
	ATP6_pos2_ND1_pos2_ND4L_pos2	TVM+F+R3	GTR+F+I+G4
	ATP6_pos3_ATP8_pos3	TIM2+F+I+G4	GTR+F+I+G4
	ATP8_pos1_ATP8_pos2	TIM2+F+I+G4	GTR+F+I+G4
	COX1_pos1	TIM2e+I+G4	SYM+I+G4
	COX1_pos2_COX2_pos2	TVM+F+I+G4	GTR+F+I+G4
	COX1_pos3	GTR+F+R4	GTR+F+I+G4
	COX2_pos1_COX3_pos1	SYM+R3	SYM+I+G4
	COX2_pos3_COX3_pos3_ND3_pos3	TIM2+F+R5	HKY+F+I+G4
	COX3_pos2	K3Pu+F+I+G4	HKY+F+I+G4
	CYTB_pos1_ND4_pos1_ND5_pos1	GTR+F+R4	GTR+F+I+G4
	CYTB_pos2_ND4_pos2	GTR+F+R3	GTR+F+I+G4
	CYTB_pos3	GTR+F+R4	GTR+F+I+G4
	ND1_pos3_ND2_pos3	TIM2+F+R4	GTR+F+I+G4
	ND2_pos1	GTR+F+I+G4	GTR+F+I+G4
	ND2_pos2_ND3_pos2	GTR+F+R3	GTR+F+I+G4
	ND4_pos3_ND4L_pos3	TIM2+F+R5	GTR+F+I+G4
	ND5_pos2	GTR+F+I+G4	GTR+F+I+G4
	ND5_pos3	TIM2+F+R5	GTR+F+I+G4
	ND6_pos1	TIM2+F+I+G4	GTR+F+I+G4
	ND6_pos2	TVM+F+I+G4	HKY+F+I+G4
	ND6_pos3	TIM3+F+I+G4	GTR+F+I+G4
	12S	GTR+F+R5	GTR+F+I+G4
	16S	GTR+F+R4	GTR+F+I+G4
PS07	ATP6_pos1_pos2_ND1_pos1_pos2_ND3_pos1_pos2	GTR+F+I+G4	GTR+F+I+G4
	ATP6_pos3_ATP8_pos3	TIM2+F+I+G4	GTR+F+I+G4
	ATP8_pos1_pos2	TIM2+F+I+G4	GTR+F+I+G4
	COX1_pos1_pos2	TIM2+F+I+G4	GTR+F+I+G4
	COX1_pos3	GTR+F+R4	GTR+F+I+G4
	COX2_pos1_pos2_COX3_pos1_pos2	GTR+F+R3	GTR+F+I+G4
	COX2_pos3_COX3_pos3_ND3_pos3	TIM2+F+R5	HKY+F+I+G4
	CYTB_pos1_pos2_ND4L_pos1_pos2_ND5_pos1_pos2	TIM2+F+R4	GTR+F+I+G4
	CYTB_pos3	GTR+F+R4	GTR+F+I+G4
	ND1_pos3_ND2_pos3	TIM2+F+R4	GTR+F+I+G4
	ND2_pos1_pos2_ND4_pos1_pos2	TIM2+F+R4	GTR+F+I+G4
	ND4_pos3_ND4L_pos3	TIM2+F+R5	GTR+F+I+G4
	ND5_pos3	TIM2+F+I+G4	GTR+F+I+G4
	ND6_pos1_pos2	GTR+F+R4	GTR+F+I+G4
	ND6_pos3	TIM3+F+I+G4	GTR+F+I+G4
	12S	GTR+F+R5	GTR+F+I+G4
	16S	GTR+F+R4	GTR+F+I+G4
PS08	ATP6_pos1_ND1_pos1	TIM2+F+I+G4	GTR+F+I+G4
	ATP6_pos3_ATP8_pos3	TIM2+F+I+G4	GTR+F+I+G4
	ATP8_pos1	TN+F+G4	GTR+F+G4
	COX1_pos1	TIM2e+I+G4	SYM+I+G4
	COX1_pos3	GTR+F+R4	GTR+F+I+G4
	COX2_pos1_COX3_pos1_ND3_pos1	SYM+I+G4	SYM+I+G4
	COX2_pos3_COX3_pos3_ND3_pos3	TIM2+F+R5	HKY+F+I+G4
	CYTB_pos1_ND4_pos1_ND4L_pos1_ND5_pos1	GTR+F+R4	GTR+F+I+G4
	CYTB_pos3_ND1_pos3_ND2_pos3	TIM2+F+R4	GTR+F+I+G4
	ND2_pos1	GTR+F+I+G4	GTR+F+I+G4
	ND4_pos3_ND4L_pos3_ND5_pos3	GTR+F+R6	GTR+F+I+G4
	ND6_pos1	TIM2+F+I+G4	GTR+F+I+G4
	ND6_pos3	TIM3+F+I+G4	GTR+F+I+G4
	12S	GTR+F+R5	GTR+F+I+G4
	16S	GTR+F+R4	GTR+F+I+G4

**[Tip 6]** You can repeat the above process using the BIC to compare the AICc results. Our comparison revealed that AICc yields an increased number of partitions; however, the models of substitution are similar across partitions. AICc also finds more parameter-rich models, particularly for the rate (R) model. In our dataset, tree topologies constructed with BIC are the same as those constructed with AICc, with minor variations in bootstrap and posterior probability values.

4. Construct ML trees for each partitioning scheme.

a. Use the substitution models indicated in *(PS01-PS08)_best_model.nex* files for each partitioning scheme. Use the nearest neighbour interchange (NNI) approach to search for tree topology. Compute branch supports with 1,000 replicates of the Shimodaira–Hasegawa approximate likelihood-ratio test (SH-aLRT; [64]) and the ultrafast bootstrapping (UFBoot2) approach [65]. Adjust these parameters accordingly to suit your dataset and computational/time constraints. Longer, more complex alignments with more sequences included will increase the computational burden.

*# Dataset 1: 13PCGs_NT*

iqtree2 -s Data_14_Galeomorphii_13PCGs_NT.fasta -st DNA -p PS01/PS01_run01_mf.best_model.nex -pre PS01/PS01_run02_ml -T 3 -B 1000 -alrt 1000

iqtree2 -s Data_14_Galeomorphii_13PCGs_NT.fasta -st DNA -p PS05/PS05_run01_mf.best_model.nex -pre PS05/PS05_run02_ml -T 3 -B 1000 -alert 1000

*# Dataset 2: 13PCGs_2rRNAs_NT*

iqtree2 -s Data_17_Galeomorphii_13PCGs_2rRNAs_NT.fasta -st DNA -p PS01/PS01_run01_mf.best_model.nex -pre PS01/PS01_run02_ml -T 3 -B 1000 -alrt 1000

iqtree2 -s Data_17_Galeomorphii_13PCGs_2rRNAs_NT.fasta -st DNA -p PS02/PS02_run01_mf.best_model.nex -pre PS02/PS02_run02_ml -T 3 -B 1000 -alrt 1000

iqtree2 -s Data_17_Galeomorphii_13PCGs_2rRNAs_NT.fasta -st DNA -p PS03/PS03_run01_mf.best_model.nex -pre PS03/PS03_run02_ml -T 3 -B 1000 -alrt 1000

iqtree2 -s Data_17_Galeomorphii_13PCGs_2rRNAs_NT.fasta -st DNA -p PS04/PS04_run01_mf.best_model.nex -pre PS04/PS04_run02_ml -T 3 -B 1000 -alrt 1000

iqtree2 -s Data_17_Galeomorphii_13PCGs_2rRNAs_NT.fasta -st DNA -p PS05/PS05_run01_mf.best_model.nex -pre PS05/PS05_run02_ml -T 3 -B 1000 -alrt 1000

iqtree2 -s Data_17_Galeomorphii_13PCGs_2rRNAs_NT.fasta -st DNA -p PS06/PS06_run01_mf.best_model.nex -pre PS06/PS06_run02_ml -T 3 -B 1000 -alrt 1000

iqtree2 -s Data_17_Galeomorphii_13PCGs_2rRNAs_NT.fasta -st DNA -p PS07/PS07_run01_mf.best_model.nex -pre PS07/PS07_run02_ml -T 3 -B 1000 -alrt 1000

iqtree2 -s Data_17_Galeomorphii_13PCGs_2rRNAs_NT.fasta -st DNA -p PS08/PS08_run01_mf.best_model.nex -pre PS08/PS08_run02_ml -T 3 -B 1000 -alrt 1000

*# Dataset 3: 13PCGs_AA*

iqtree2 -s Data_20_Galeomorphii_13PCGs_AA.fasta -st AA -p PS01/PS01AA_run01_mf.best_model.nex -pre PS01/PS01AA_run02_ml -T 3 -B 1000 -alrt 1000

iqtree2 -s Data_20_Galeomorphii_13PCGs_AA.fasta -st AA -p PS05/PS05AA_run01_mf.best_model.nex -pre PS05/PS05AA_run02_ml -T 3 -B 1000 -alrt 1000

5. Investigate topological conflict around each branch of the species tree by calculating gene and site concordance factors in IQ-Tree.

a. Infer concatenation-based species trees with 1,000 ultrafast bootstraps and an edge-linked partition model. Use the *(PS01-PS08)_run01_mf.best_scheme.nex* files as input partition files.

*# Dataset 1: 13PCGs_NT*

## Calculate gene concordance factors (gCF).

iqtree2 -s Data_14_Galeomorphii_13PCGs_NT.fasta -p PS01/PS01_run01_mf.best_scheme.nex --prefix PS01/PS01_run03_concat.condonpart.MF -B 1000 -T 3

iqtree2 -s Data_14_Galeomorphii_13PCGs_NT.fasta -p PS05/PS05_run01_mf.best_scheme.nex --prefix PS05/PS05_run03_concat.condonpart.MF -B 1000 -T 3

## Calculate site concordance factors (sCF) and infer the locus trees.

iqtree2 -s Data_14_Galeomorphii_13PCGs_NT.fasta -S PS01/PS01_run01_mf.best_scheme.nex --prefix PS01/PS01_run03_loci.condonpart.MF -T 3

iqtree2 -s Data_14_Galeomorphii_13PCGs_NT.fasta -S PS05/PS05_run01_mf.best_scheme.nex --prefix PS05/PS05_run03_loci.condonpart.MF -T 3

## Compute concordance factors.

iqtree2 -t PS01/PS01_run03_concat.condonpart.MF.treefile --gcf PS01/PS01_run03_loci.condonpart.MF.treefile -s 13PCGs_NT.fasta --scf 100 -seed 471990 --prefix PS01/PS01_run03_concord

iqtree2 -t PS05/PS05_run03_concat.condonpart.MF.treefile --gcf PS05/PS05_run03_loci.condonpart.MF.treefile -s 13PCGs_NT.fasta --scf 100 -seed 471990 --prefix PS05/PS05_run03_concord

*# Dataset 2: 13PCGs_2rRNAs_NT*

## Calculate gene concordance factors (gCF).

iqtree2 -s Data_17_Galeomorphii_13PCGs_2rRNAs_NT.fasta -p PS01/PS01_run01_mf.best_scheme.nex --prefix PS01/PS01_run03_concat.condonpart.MF -B 1000 -T 3

iqtree2 -s Data_17_Galeomorphii_13PCGs_2rRNAs_NT.fasta -p PS02/PS02_run01_mf.best_scheme.nex --prefix PS02/PS02_run03_concat.condonpart.MF -B 1000 -T 3

iqtree2 -s Data_17_Galeomorphii_13PCGs_2rRNAs_NT.fasta -p PS03/PS03_run01_mf.best_scheme.nex --prefix PS03/PS03_run03_concat.condonpart.MF -B 1000 -T 3

iqtree2 -s Data_17_Galeomorphii_13PCGs_2rRNAs_NT.fasta -p PS04/PS04_run01_mf.best_scheme.nex --prefix PS04/PS04_run03_concat.condonpart.MF -B 1000 -T 3

iqtree2 -s Data_17_Galeomorphii_13PCGs_2rRNAs_NT.fasta -p PS05/PS05_run01_mf.best_scheme.nex --prefix PS05/PS05_run03_concat.condonpart.MF -B 1000 -T 3

iqtree2 -s Data_17_Galeomorphii_13PCGs_2rRNAs_NT.fasta -p PS06/PS06_run01_mf.best_scheme.nex --prefix PS06/PS06_run03_concat.condonpart.MF -B 1000 -T 3

iqtree2 -s Data_17_Galeomorphii_13PCGs_2rRNAs_NT.fasta -p PS07/PS07_run01_mf.best_scheme.nex --prefix PS07/PS07_run03_concat.condonpart.MF -B 1000 -T 3

iqtree2 -s Data_17_Galeomorphii_13PCGs_2rRNAs_NT.fasta -p PS08/PS08_run01_mf.best_scheme.nex --prefix PS08/PS08_run03_concat.condonpart.MF -B 1000 -T 3

## Calculate site concordance factors (sCF) and infer the locus trees.

iqtree2 -s Data_17_Galeomorphii_13PCGs_2rRNAs_NT.fasta -S PS01/PS01_run01_mf.best_scheme.nex --prefix PS01/PS01_run03_loci.condonpart.MF -T 3

iqtree2 -s Data_17_Galeomorphii_Data_17_Galeomorphii_13PCGs_2rRNAs_NT.fasta -S PS02/PS02_run01_mf.best_scheme.nex --prefix PS02/PS02_run03_loci.condonpart.MF -T 3

iqtree2 -s Data_17_Galeomorphii_13PCGs_2rRNAs_NT.fasta -S PS03/PS03_run01_mf.best_scheme.nex --prefix PS03/PS03_run03_loci.condonpart.MF -T 3

iqtree2 -s Data_17_Galeomorphii_13PCGs_2rRNAs_NT.fasta -S PS04/PS04_run01_mf.best_scheme.nex --prefix PS04/PS04_run03_loci.condonpart.MF -T 3

iqtree2 -s Data_17_Galeomorphii_13PCGs_2rRNAs_NT.fasta -S PS05/PS05_run01_mf.best_scheme.nex --prefix PS05/PS05_run03_loci.condonpart.MF -T 3

iqtree2 -s Data_17_Galeomorphii_13PCGs_2rRNAs_NT.fasta -S PS06/PS06_run01_mf.best_scheme.nex --prefix PS06/PS06_run03_loci.condonpart.MF -T 3

iqtree2 -s Data_17_Galeomorphii_13PCGs_2rRNAs_NT.fasta -S PS07/PS07_run01_mf.best_scheme.nex --prefix PS07/PS07_run03_loci.condonpart.MF -T 3

iqtree2 -s Data_17_Galeomorphii_13PCGs_2rRNAs_NT.fasta -S PS08/PS08_run01_mf.best_scheme.nex --prefix PS08/PS08_run03_loci.condonpart.MF -T 3

## Compute concordance factors.

iqtree2 -t PS01/PS01_run03_concat.condonpart.MF.treefile --gcf PS01/PS01_run03_loci.condonpart.MF.treefile -s Data_17_Galeomorphii_13PCGs_2rRNAs_NT.fasta --scf 100 -seed 471990 --prefix PS01/PS01_run03_concord

iqtree2 -t PS02/PS02_run03_concat.condonpart.MF.treefile --gcf PS02/PS02_run03_loci.condonpart.MF.treefile -s Data_17_Galeomorphii_13PCGs_2rRNAs_NT.fasta --scf 100 -seed 471990 --prefix PS02/PS02_run03_concord

iqtree2 -t PS03/PS03_run03_concat.condonpart.MF.treefile --gcf PS03/PS03_run03_loci.condonpart.MF.treefile -s Data_17_Galeomorphii_13PCGs_2rRNAs_NT.fasta --scf 100 -seed 471990 --prefix PS03/PS03_run03_concord

iqtree2 -t PS04/PS04_run03_concat.condonpart.MF.treefile --gcf PS04/PS04_run03_loci.condonpart.MF.treefile -s Data_17_Galeomorphii_13PCGs_2rRNAs_NT.fasta --scf 100 -seed 471990 --prefix PS04/PS04_run03_concord

iqtree2 -t PS05/PS05_run03_concat.condonpart.MF.treefile --gcf PS05/PS05_run03_loci.condonpart.MF.treefile -s Data_17_Galeomorphii_13PCGs_2rRNAs_NT.fasta --scf 100 -seed 471990 --prefix PS05/PS05_run03_concord

iqtree2 -t PS06/PS06_run03_concat.condonpart.MF.treefile --gcf PS06/PS06_run03_loci.condonpart.MF.treefile -s Data_17_Galeomorphii_13PCGs_2rRNAs_NT.fasta --scf 100 -seed 471990 --prefix PS06/PS06_run03_concord

iqtree2 -t PS07/PS07_run03_concat.condonpart.MF.treefile --gcf PS07/PS07_run03_loci.condonpart.MF.treefile -s Data_17_Galeomorphii_13PCGs_2rRNAs_NT.fasta --scf 100 -seed 471990 --prefix PS07/PS07_run03_concord

iqtree2 -t PS08/PS08_run03_concat.condonpart.MF.treefile --gcf PS08/PS08_run03_loci.condonpart.MF.treefile -s Data_17_Galeomorphii_13PCGs_2rRNAs_NT.fasta --scf 100 -seed 471990 --prefix PS08/PS08_run03_concord

*# Dataset 3: 13PCGs_AA*

## Calculate gene concordance factors (gCF).

iqtree2 -s Data_20_Galeomorphii_13PCGs_AA.fasta -p PS01/PS01AA_run01_mf.best_scheme.nex --prefix PS01/PS01AA_run03_concat.condonpart.MF -B 1000 -T 3

iqtree2 -s Data_20_Galeomorphii_13PCGs_AA.fasta -p PS05/PS05AA_run01_mf.best_scheme.nex --prefix PS05/PS05AA_run03_concat.condonpart.MF -B 1000 -T 3

## Calculate site concordance factors (sCF) and infer the locus trees.

iqtree2 -s Data_20_Galeomorphii_13PCGs_AA.fasta -S PS01/PS01AA_run01_mf.best_scheme.nex --prefix PS01/PS01AA_run03_loci.condonpart.MF -T 3

iqtree2 -s Data_20_Galeomorphii_13PCGs_AA.fasta -S PS05/PS05AA_run01_mf.best_scheme.nex --prefix PS05/PS05AA_run03_loci.condonpart.MF -T 3

## Compute concordance factors.

iqtree2 -t PS01/PS01AA_run03_concat.condonpart.MF.treefile --gcf PS01/PS01AA_run03_loci.condonpart.MF.treefile -s Data_20_Galeomorphii_13PCGs_AA.fasta --scf 100 -seed 471990 --prefix PS01/PS01AA_run03_concord

iqtree2 -t PS05/PS05AA_run03_concat.condonpart.MF.treefile --gcf PS05/PS05AA_run03_loci.condonpart.MF.treefile -s Data_20_Galeomorphii_13PCGs_AA.fasta --scf 100 -seed 471990 --prefix PS05/PS05AA_run03_concord

6. Compare the trees from the eight runs to determine significant diﬀerences with the approximately unbiased (AU) tree topology test [59] also implemented in IQ-Tree (Table 5).

a. Paste the contents of the *(PS01-PS08)_run02_ML.treefiles* in Notepad and save as a list in newick format (*TopoTest_PS01-PS08.treesls*) to use as the input file.

b. Use *-z* to compute the log-likelihood.

c. Set the number of search iterations (*-n*) to 0.

d. Run tree topology tests using the RELL approximation [66]. *-zb* specifies the number of RELL replicates.

e. Perform weighted KH and weighted SH tests (*-zw*).

f. Conduct an approximately unbiased (*-au*) test [59].

iqtree2 -s Data_14_Galeomorphii_13PCGs_NT.fasta -z TopoTest_PS01-PS08.treesls --prefix TopoTest_run01 -n 0 -zb 10000 -zw -au -nt AUTO

iqtree2 -s Data_17_Galeomorphii_13PCGs_2rRNAs_NT.fasta -z TopoTest_PS01-PS08.treesls --prefix TopoTest_run01 -n 0 -zb 10000 -zw -au -nt AUTO

**Table 5.** Comparison of the log-likelihood values to assess the confidence of maximum likelihood tree selection using the eight partitioning schemes of Dataset 1: 13PCGs_NT. Tree topology tests were run using 10,000 RELL replicates as implemented in IQ-Tree v.2.2.0.3 [45]. RELL, resampling of estimated log-likelihoods.

Tree	logL	deltaL	bp-RELL		p-KH		p-SH		p-WKH		p-WSH		c-ELW		p-AU	
PS01	-208906	0	0.525	+	0.641	+	1	+	0.641	+	0.851	+	0.521	+	0.708	+
PS02	-208914	7.8506	0.109	+	0.359	+	0.734	+	0.359	+	0.847	+	0.115	+	0.536	+
PS03	-208914	7.8506	0.112	+	0.359	+	0.734	+	0.359	+	0.825	+	0.115	+	0.535	+
PS04	-208933	27.449	0.051	+	0.172	+	0.324	+	0.163	+	0.395	+	0.0493	+	0.213	+
PS05	-208919	13.672	0.121	+	0.275	+	0.605	+	0.275	+	0.624	+	0.117	+	0.378	+
PS06	-208942	36.242	0.023	-	0.151	+	0.21	+	0.151	+	0.464	+	0.022	-	0.182	+
PS07	-208939	33.133	0.0399	+	0.16	+	0.235	+	0.16	+	0.43	+	0.0393	+	0.208	+
PS08	-208942	36.242	0.0194	-	0.151	+	0.21	+	0.151	+	0.476	+	0.022	-	0.182	+

deltaL: logL difference from the maximal logL in the set.

bp-RELL: Bootstrap proportion using the RELL method [66].

p-KH: p-value of one-sided Kishino–Hasegawa test [67].

p-SH: p-value of Shimodaira–Hasegawa test [68].

p-WKH: p-value of weighted KH test.

p-WSH: p-value of weighted SH test.

c-ELW: Expected likelihood weight of Strimmer & Rambaut [69].

p-AU: p-value of approximately unbiased (AU) test [59].

Plus signs denote 95% confidence sets.

Minus signs denote significant exclusion.

**[Tip 7]** A tree is rejected if its p-value is <0.05 (marked with a - sign) for KH, SH, and AU tests. bp-RELL and c-ELW return posterior weights that are not p-values. The weights sum up to 1 across the evaluated trees. The tests here presented returned non-significant p-values (p > 0.05), and log-likelihood values are comparable among partitioning schemes. PS06 and PS08 have significant posterior weights for the bp-RELL method and c-ELW, likely attributable to overpartitioning for these partitioning schemes.

7. Conduct a χ2-test to determine whether the frequency of gene trees (gCF) and sites (sCF) supporting the two alternative topologies differ significantly (the hypothesis of equal frequencies) and construct plots comparing gCF and sCF with UFBoot2 values as implemented in Lanfear’s R script [60]. See https://www.robertlanfear.com/blog/files/concordance_factors.html (last accessed 1/12/2025) for a tutorial. Here, we used PS05 to demonstrate the resulting plots and statistics produced when running the script, but we ran it on all partitioning schemes.

# Adapted from Lanfear’s R script (Minh et al., 2020).

library(viridis)

library(ggplot2)

library(dplyr)

library(ggrepel)

library(GGally)

library(entropy)

# Read the data

PS05 = read.delim(‘./PS05_run03_concord.cf.stat’, header = T, comment.char = '#')

names(PS05)[18] = "bootstrap"

names(PS05)[19] = "branchlength"

# Plot the relationship between concordance factors and bootstrap values (Figure 9)

pdfPath <- './CF plots/PS05/'

pdf(paste(pdfPath, '/CF_plots_PS05.pdf', sep=''), width=8, height=6)

ggplot(PS05, aes(x = gCF, y = sCF)) +

 geom_point(aes(colour = bootstrap)) +

 scale_colour_viridis(direction = -1) +

 xlim(0, 100) +

 ylim(0, 100) +

 geom_abline(slope = 1, intercept = 0, linetype = "dashed")

dev.off()

# Use the concordance factors to test the assumptions of an ILS model

chisq = function(DF1, DF2, N){

 tryCatch({

 # converts percentages to counts, runs chisq, gets pvalue

 chisq.test(c(round(DF1*N)/100, round(DF2*N)/100))$p.value

 },

 error = function(err) {

 # errors come if you give chisq two zeros

 # but here we're sure that there's no difference

 return(1.0)

 })

}

e = PS05 %>%

 group_by(ID) %>%

 mutate(gEF_p = chisq(gDF1, gDF2, gN)) %>%

 mutate(sEF_p = chisq(sDF1, sDF2, sN))

subset(data.frame(e), (gEF_p < 0.05 | sEF_p < 0.05))

write_xlsx(e, ‘./CF plots/PS05/e.xlsx’)

# Calculate the internode certainty

IC = function(CF, DF1, DF2, N){

 # convert to counts

 X = CF * N / 100

 Y = max(DF1, DF2) * N / 100

 pX = X/(X+Y)

 pY = Y/(X+Y)

 IC = 1 + pX * log2(pX) +

 pY * log2(pY)

 return(IC)

}

e = e %>%

 group_by(ID) %>%

 mutate(gIC = IC(gCF, gDF1, gDF2, gN)) %>%

 mutate(sIC = IC(sCF, sDF1, sDF2, sN))

ENT = function(CF, DF1, DF2, N){

 CF = CF * N / 100

 DF1 = DF1 * N / 100

 DF2 = DF2 * N / 100

 return(entropy(c(CF, DF1, DF2)))

}

ENTC = function(CF, DF1, DF2, N){

 maxent = 1.098612

 CF = CF * N / 100

 DF1 = DF1 * N / 100

 DF2 = DF2 * N / 100

 ent = entropy(c(CF, DF1, DF2))

 entc = 1 - (ent / maxent)

 return(entc)

}

e = e %>%

 group_by(ID) %>%

 mutate(sENT = ENT(sCF, sDF1, sDF2, sN)) %>%

 mutate(sENTC = ENTC(sCF, sDF1, sDF2, sN))

# Plot internode certainty (Figure 10)

pdf(‘./internode_certainty_plot.pdf’)

ggpairs(e, columns = c(2, 6, 10, 12, 13, 14, 15, 16, 17))

dev.off()

**Figure 9.**
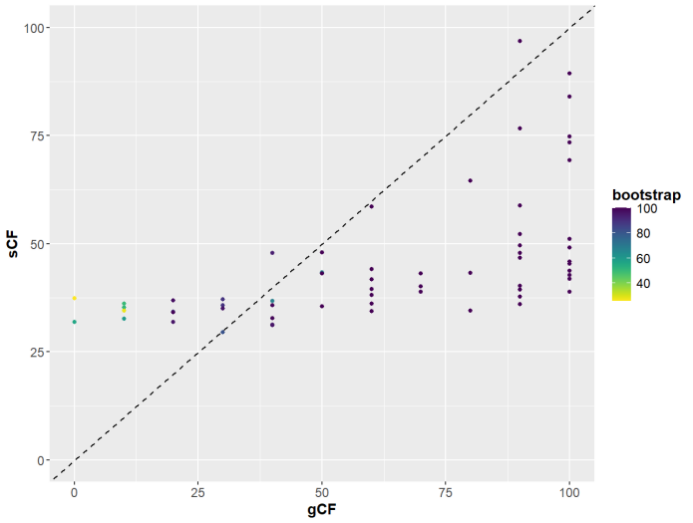
Relationship between gene and site concordance factors (gCF and sCF) and bootstraps (UFBoot2) for partition scheme 5 (PS05: 13 PCGs + 2 rRNAs, 15 partitions) created using Lanfear’s R script [60] in R v.4.1.2 [70]. X-axis: gCF values; y-axis: sCF values; legend: bootstrap values shown as a gradient with deep purple being high and yellow being low.

**Figure 10.**
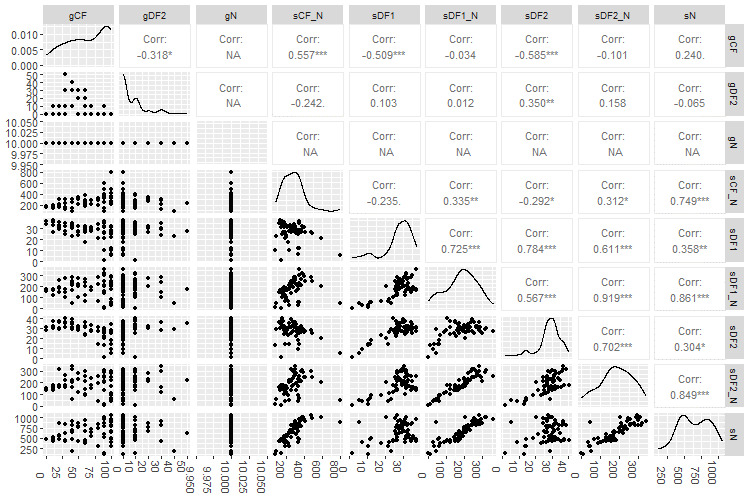
Internode certainty plot comparing the extent to which concordance factors, internode certainty, entropy, and bootstrap values differ from each other for partition scheme 5 (PS05: 13 PCGs + 2 rRNAs, 15 partitions) created using Lanfear’s R script [60] in R v.4.1.2. For our study, we focused on comparing differences between bootstrap support and concordance factor values. Probability values less than 5% (p < 0.05) is the recommended threshold for significance. gCF: gene concordance factor; gDF2: gene discordance factor 2; gN: number of single locus trees containing a specific branch; sCF_N: site concordance factor number; sDF1: site discordance factor 1; sN: number of decisive sites for a specific branch; sDF1_N: site discordance factor 1 multiplied by sN; sDF2: site discordance factor 2; sDF2_N: site discordance factor 2 number multiplied by sN.

a. From the output files, use “e” to construct a table of Chi-squared values comparing gCFs and sCFs for the different partitioning schemes (Table 6).

**Table 6.** Chi-squared (χ2) test to see if the frequency of gene trees (gCF) and sites (sCF) supporting the two alternative topologies differed significantly for some branches of the phylogeny constructed using partitioning scheme 5 (PS05: 13PCGs + 2 rRNAs, 15 partitions) as implemented in Lanfear’s R script [60] (http://www.robertlanfear.com/blog/files/concordance_factors.html) in R v.4.1.2. Focus on the last two columns, which show the probability that the data can reject equal frequencies for genes (gEFp) and for sites (sEFp). The significance threshold for probability values is 5% (p < 0.05). It is important to flag that the χ^2^-approach is not accurate among sites in a single gene because of linkage disequilibrium, so sEF p-values must be interpreted cautiously. ID: branch identification number; gCF: gene concordance factor; gDF2: gene discordance factor 2; gN: number of single locus trees containing a specific branch; sCF_N: site concordance factor number; sDF1: site discordance factor 1; sN: number of decisive sites for a specific branch; sDF1_N: site discordance factor 1 multiplied by sN; sDF2: site discordance factor 2; sDF2_N: site discordance factor 2 number multiplied by sN.

ID	gCF	gCF_N	gDF1	gDF1_N	gDF2	gDF2_N	gDFP	gDFP_N	gN	sCF	sCF_N	sDF1	sDF1_N	sDF2	sDF2_N	sN	bootstrap	branchlength	gEF_p	sEF_p
65	70	7	10	1	10	1	10	1	10	38.84	276.13	27.65	196.6	33.5	238.21	710.94	100	0.016703	1	0.046075
66	90	9	10	1	0	0	0	0	10	58.77	585.6	20.78	206.6	20.45	203.46	995.66	100	0.138318	0.317311	0.871006
67	90	9	0	0	0	0	10	1	10	35.96	343.79	37.18	356.38	26.86	256.98	957.15	100	0.051834	1	6.61E-05
68	40	4	10	1	30	3	20	2	10	32.72	299.31	30.72	282.21	36.56	336.91	918.43	99	0.023262	0.317311	0.030939
69	20	2	30	3	0	0	50	5	10	34.24	297.24	29.32	253.49	36.44	316.64	867.37	84	0.008597	0.083265	0.00971
70	90	9	0	0	0	0	10	1	10	40.24	343.09	34.97	298.88	24.79	211.73	853.7	100	0.065055	1	0.000119
71	50	5	10	1	20	2	20	2	10	43.33	330.61	26.37	200.85	30.3	230.23	761.69	67	0.008898	0.563703	0.1497
72	40	4	20	2	10	1	30	3	10	36.77	238.84	32.3	209.31	30.93	200.83	648.98	68	0.008431	0.563703	0.660764
73	90	9	0	0	0	0	10	1	10	52.21	314.19	25.9	155.92	21.88	131.72	601.83	100	0.035249	1	0.153717
74	50	5	20	2	30	3	0	0	10	48.04	203.04	24.26	102.39	27.7	117.05	422.48	100	0.008632	0.654721	0.326416
75	100	10	0	0	0	0	0	0	10	74.78	236.5	10.56	33.38	14.66	46.29	316.17	100	0.023451	1	0.146687
76	90	9	10	1	0	0	0	0	10	76.7	86.03	10.34	11.61	12.96	14.52	112.16	100	0.005371	0.317311	0.565267
77	100	10	0	0	0	0	0	0	10	73.45	348.07	11.91	56.33	14.63	69.19	473.59	100	0.044457	1	0.250248
78	40	4	20	2	40	4	0	0	10	47.91	92.38	23.36	45.13	28.73	55.33	192.84	94	0.003143	0.414216	0.301754
79	80	8	0	0	0	0	20	2	10	34.47	285.89	34.5	286.3	31.03	257.93	830.12	100	0.02766	1	0.2169
80	30	3	30	3	0	0	40	4	10	35.79	298.95	32.65	272.63	31.56	263.28	834.86	89	0.0085	0.083265	0.694291
81	60	6	0	0	10	1	30	3	10	36.16	292.19	32.38	261.84	31.46	254.47	808.5	100	0.013043	0.317311	0.743302
82	40	4	20	2	10	1	30	3	10	31.3	254.73	30.13	244.92	38.57	314.97	814.62	94	0.00751	0.563703	0.003659
83	100	10	0	0	0	0	0	0	10	38.85	318.14	26.67	218.41	34.49	282.3	818.85	100	0.058356	1	0.004221
84	100	10	0	0	0	0	0	0	10	89.36	797.31	5.34	47.25	5.3	47.14	891.7	100	0.161267	1	0.970518
85	90	9	0	0	10	1	0	0	10	96.89	144.43	1.52	2.29	1.59	2.37	149.09	100	0.012856	0.317311	0.962972
86	90	9	0	0	0	0	10	1	10	46.72	371.16	26.62	211.05	26.66	211.38	793.59	100	0.026811	1	0.987584
87	70	7	0	0	0	0	30	3	10	43.18	279.63	29.44	191.09	27.37	177.82	648.54	100	0.017242	1	0.48446
88	20	2	10	1	10	1	60	6	10	36.88	206.32	33.54	188.1	29.58	166.12	560.54	97	0.006179	1	0.237914
89	10	1	0	0	0	0	90	9	10	35.25	185.11	32.82	171.54	31.93	168.43	525.08	51	0.003887	1	0.80006
90	10	1	0	0	0	0	90	9	10	34.47	169.78	35.7	177.39	29.83	148.58	495.75	28	0.00394	1	0.106414
91	0	0	10	1	0	0	90	9	10	37.42	175.61	34.42	161.86	28.16	131.17	468.64	26	0.001746	0.317311	0.086668

8. Perform Bayesian inference analyses via the CIPRES Gateway (https://www.phylo.org/) or your high-performance computer of choice.

a. Create a new folder titled *2_MrBayes_Runs* in the directory *4b_BI*. Make *13_PCGs_2rRNAs* and *13PCGs* folders as conducted previously. Create nexus files for each partitioning scheme of Dataset 2 containing the full alignment in nexus format (Data 19 in the sample files) as well as the best-fit partitioning schemes and models of evolution determined by IQ-Tree in step C2 above and save them in the folder *13_PCGs_2rRNAs*. Do the same for Datasets 1 and 3 (Data 17 and 22) and save the nexus files in the *13PCGs* folder. Below is an example of the nexus data blocks with partitions and evolutionary models included after the alignment for PS05.

begin mrbayes;

charset ATP6 = 1 - 681;

charset ATP8 = 682 - 846;

charset COX1 = 847 - 2400;

charset COX2 = 2401 - 3090;

charset COX3_ND3 = 3091 - 3873 7036 - 7383;

charset CYTB_ND1_ND4_ND4L_ND5 = 3874 - 5016 5017 - 5988 7384 - 8763 8764 - 9057 9058 - 10884;

charset ND2 = 5989 - 7035;

charset ND6 = 10885 - 11403;

charset 12S = 11404 - 12329;

charset 16S = 12330 - 13945;

partition iqtree = 10: ATP6, ATP8, COX1, COX2, COX3_ND3, CYTB_ND1_ND4_ND4L_ND5, ND2, ND6, 12S, 16S;

 set partition = iqtree;

set autoclose=yes nowarn=yes;

lset applyto=(1,2,3,4,3,4,5,6,7,8,9,10) nst=6 rates=invgamma;

mcmc ngen=1500000 relburnin=yes burninfrac=0.25 samplefreq=1000 printfreq=10000 Nruns=2 nchains=4 savebrlens=yes;

sump burnin=375;

sumt burnin=375;

 end;

b. Run a pair of independent searches for 5 million generations, with trees saved every 1,000 generations and the first 2,500 sampled trees of each search discarded as burn-in for each partitioning scheme of each dataset.

for g in *.nex

do

mb -i $g

done

c. Screen the model parameter summary statistics “estimated sample size” (ESS) and “potential scale reduction factor” (PSRF), where convergence occurred at ESS >200 and PSRF ~1.0, in R. Download the source code file *plot_mrb* from https://rdrr.io/github/kmiddleton/kmmisc/man/plot_mrb.html (last accessed 1/12/2025).

# Install

remotes::install_github("stan-dev/cmdstanr")

options(repos = c(

 yihui = 'https://yihui.r-universe.dev',

 CRAN = 'https://cloud.r-project.org'

))

install.packages('xfun')

# Load pakages and source file

library(xfun) # for file_ext() function

library(ggplot2) # for fggplot() function

library(scales) # for scientific_format() function

source("./4b_BI/2_mb_runs/plot_mrb.R")

# Run

plot_mrb()

**Figure 11.**
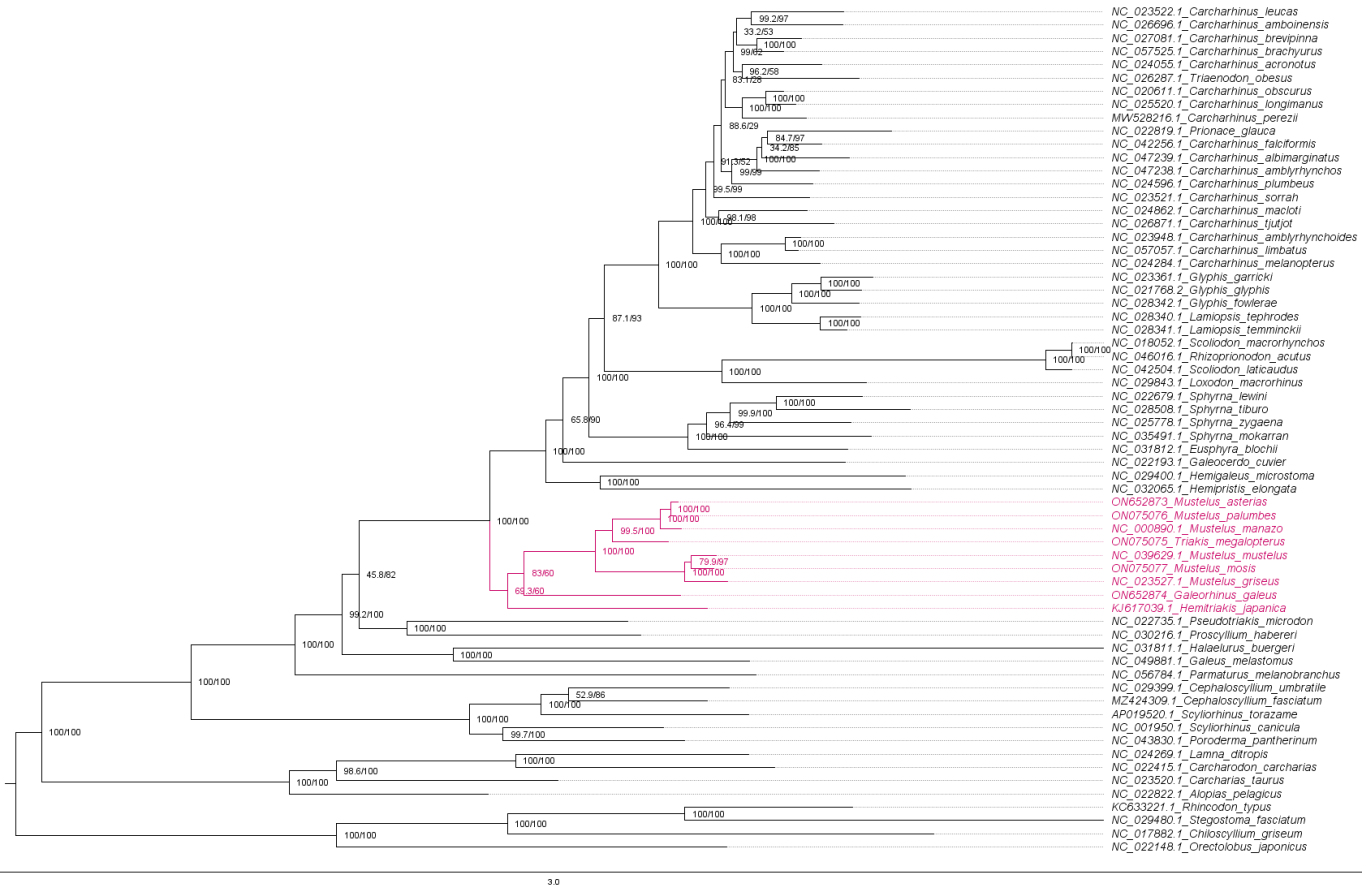
Maximum likelihood phylogeny of species in the order Carcharhiniformes (with representative Lamniform and Orectolobiform outgroups) constructed using 13 protein-coding genes and 2 rRNA genes with the PS05 gene partitioning scheme (13 PCGs + 2 rRNAs; 15 partitions) visualised in FigTree v.1.4.4 [47]. Nodes with UFBoot2 ≥ 95 and SH-aLRT ≥ 80 are considered well-supported [45].

**Figure 12.**
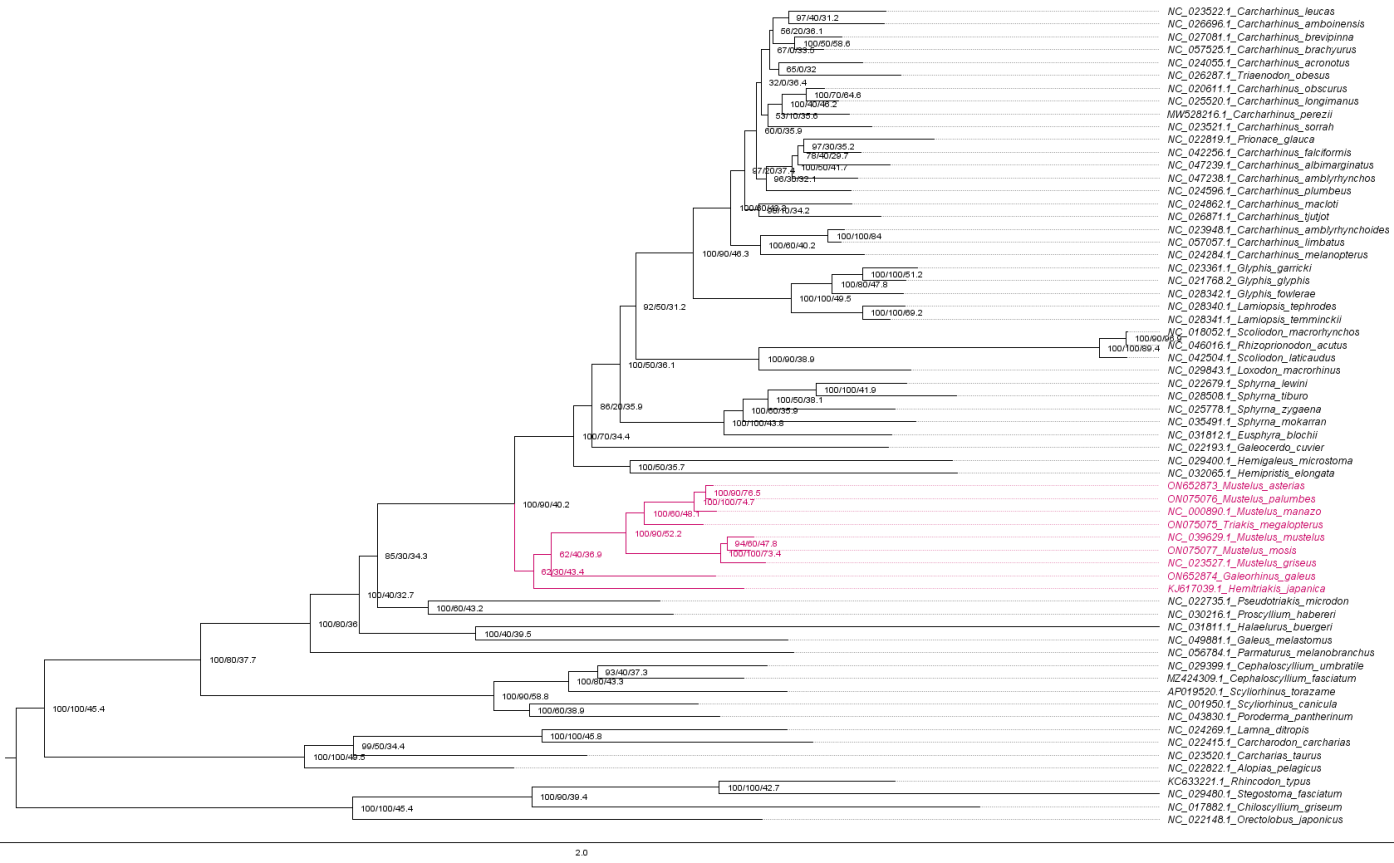
Maximum likelihood phylogeny with gene and site concordance factors (gCF and sCF) for the PS05 gene partitioning scheme (13 PCGs + 2 rRNAs; 15 partitions) visualised in FigTree v.1.4.4 [47]. Nodes with sCF values below 33% and gCF values that are lower than sCF values or near zero require further attention.

**Figure 13.**
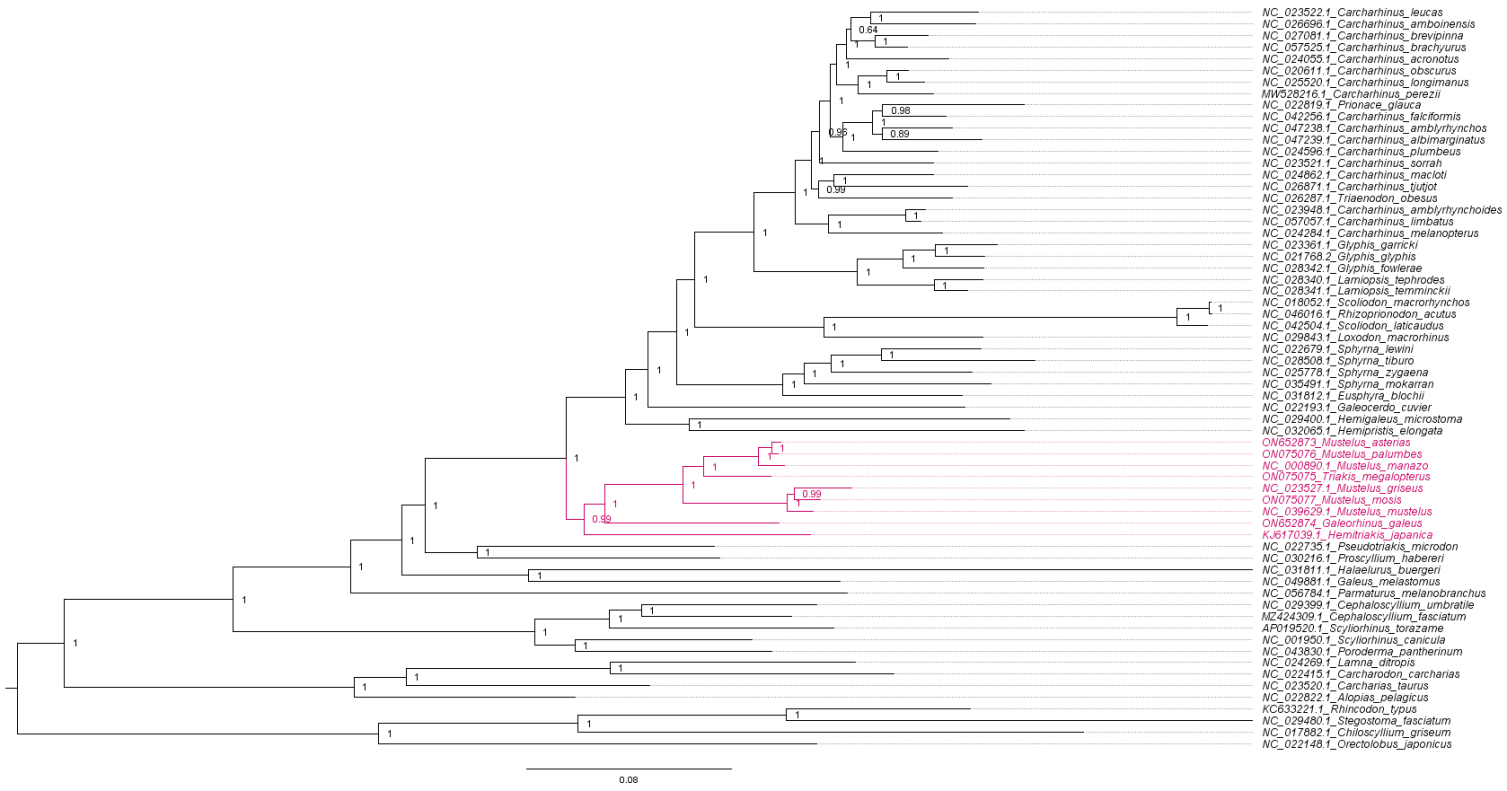
Bayesian inference phylogeny for the PS05 gene partitioning scheme (13 PCGs + 2 rRNAs; 15 partitions visualised in FigTree v.1.4.4 [47]. Nodes with posterior probability values higher than or equal to 95 are considered well-supported.

9. Visualise and compare the ML, BI, and concordance factor trees in FigTree.

a. Open all nex.treefiles from the ML and BI analyses in FigTree and root at the outgroup.

b. Align nodes and view in increasing order.

c. Colour the branches and nodes of interest or use colours to differentiate between different groups of species. Here, we coloured our focal family, the houndsharks, in pink.

d. Add bootstrap, concordance factors, and posterior probability values.

e. Save them as png files and visualise (Figures 11, 12, and 13).

**[Tip 8]** Questions to ask when comparing the trees (the interpretation of results is discussed in detail in General Note 3):

Do the deeper nodes (i.e., order of your main groups) show consistency across partitioning schemes?

How do the bootstrap support and posterior probability values of deeper nodes compare to those of shallower nodes?

How do the concordance factor values compare to the bootstrap values and to each other?

Are there any nodes with a gCF value of 0 or/and sCF value of ~33%? sCF values have a minimum value of ~30% because they are calculated by comparing the three possible resolutions of a quartet around a node, so when the data are completely equivocal about these resolutions, we expect an sCF value of 33%. However, gCF values can reach 0% if no gene tree contains a branch present in the species tree because they are calculated from full gene trees, such that there are many more than three possible resolutions around a node [60]. This can happen when a combination of biology and stochastic error leads to gene-tree discordance.

Is there evidence of polytomy in any of the groups? A polytomy is a node in a phylogenetic tree where more than two branches descend from a single ancestral lineage. This may represent several speciation events occurring simultaneously or it can indicate that there is not enough information to resolve these relationships.

How do branch lengths compare among groups and partitioning strategies?

**Figure 14.**
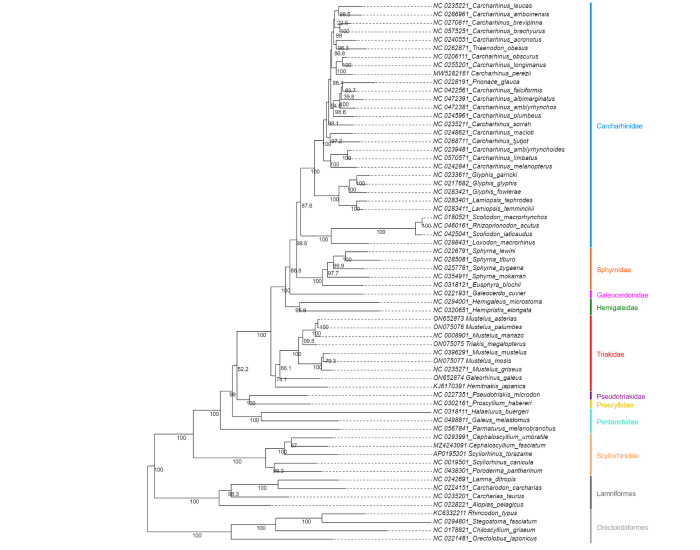
Maximum likelihood phylogeny of species in the order Carcharhiniformes (with representative Lamniform and Orectolobiform outgroups) constructed using 13 protein-coding genes and 2 rRNA genes with the PS05 gene partitioning scheme (13 PCGs + 2 rRNAs; 15 partitions) in Evolview v.3 [48]. Ultrafast bootstrap (UFBoot2) values are indicated at the nodes. Lineages are grouped into families indicated by the coloured bars.

10. Construct the annotated consensus tree (Figure 14).

a. Save the best supported trees from the ML and BI analyses (with support values) in a newick format.

b. Navigate to the Evolview v3 webpage (https://www.evolgenius.info/evolview/) and start a new project.

c. Import the newick file.

d. Adjust size and layout and select bootstrap values.

e. Import annotations. The annotations described here can be changed depending on what you want to display. The script below can be used as guidance when designing your own script file for your dataset. We designated colours to each family and order included in our study. When assigning a colour to a specific group, make sure to type the ID of the individual that appears at the top of the cluster followed by the one that appears at the bottom. Everything in between will also be designated to that group.

# Evolview Annotations

# Denoting families and outgroups

!grouplabelstyle=1

!op0.8

NC_023522.1_Carcharhinus_leucas,NC_029843.1_Loxodon_macrorhinustext=Carcharhinidae,color=#008EED,textorientation=horizontal,linewidth=4,fontsize=16

NC_022193.1_Galeocerdo_cuviertext=Galeocerdonidae, color=#FF00FF,textorientation=horizontal,linewidth=4,fontsize=16

NC_022679.1_Sphyrna_lewini,NC_031812.1_Eusphyra_blochiitext=Sphyrnidae, color=#FF6821, textorientation=horizontal,linewidth=4,fontsize=16

NC_029400.1_Hemigaleus_microstoma,NC_032065.1_Hemipristis_elongatatext=Hemigaleidae, color=green, textorientation=horizontal,linewidth=4,fontsize=16

ON652873_Mustelus_asterias,KJ617039.1_Hemitriakis_japanicatext=Triakidae,color=red, textorientation=horizontal,linewidth=4,fontsize=16

NC_022735.1_Pseudotriakis_microdontext=Pseudotriakidae, color=purple,textorientation=horizontal,linewidth=4,fontsize=16

NC_030216.1_Proscyllium_habereritext=Proscyllidae,color=#EAC400,textorientation=horizontal,linewidth=4,fontsize=16

NC_031811.1_Halaelurus_buergeri,NC_056784.1_Parmaturus_melanobranchustext=Pentanchidae,color=turquoise,textorientation=horizontal,linewidth=4,fontsize=16

NC_029399.1_Cephaloscyllium_umbratile,NC_043830.1_Poroderma_pantherinumtext=Scyliorhinidae,color=#F4A460,textorientation=horizontal,linewidth=4,fontsize=16

NC_024269.1_Lamna_ditropis,NC_022822.1_Alopias_pelagicustext=Lamniformes,color=#626469,textorientation=horizontal,linewidth=4,fontsize=16

KC633221.1_Rhincodon_typus,NC_022148.1_Orectolobus_japonicustext=Orectolobiformes,color=#A4A5A9,textorientation=horizontal,linewidth=4,fontsize=16

**D. Multispecies coalescent modelling**

After review of the different trees constructed for the eight partitioning schemes using ML and BI methods, as well as concordance factors, it was apparent that there was phylogenetic discordance in shallower lineages of our dataset, which can be ascribed to gene tree conflicts affecting species tree inference. This incomplete lineage sorting made it necessary to utilise multispecies coalescent modelling to estimate the effects of gene-tree conflict on species-tree inference. This protocol explains how to employ the summary method, Accurate Species TRee ALgorithm (ASTRAL v.5.6.3) [49], and the site-based method, SVDQuartets [50], in PAUP* v4.0a 169 [51]. ASTRAL seeks to find the tree that maximises the number of induced quartet trees in gene trees that are shared by the species tree using pre-estimated gene trees [49], whereas SVDQuartets directly infers the species tree from sequence alignments using singular value decomposition (SVD) to analyse quartets of taxa one at a time, extracting information from patterns of genetic variation across the genome [71].

Assuming that incomplete lineage sorting (ILS) is the only source of discordance between gene and species trees, the multi-species coalescent model predicts that the likelihood of a gene tree quartet matching the species tree topology is greater than the likelihood of matching the two alternatives. Furthermore, the two alternatives will have comparable frequencies [72,73]. We use our Galeomorphi data files as input for the programs. Substitute with your own data files.

Refer to this GitHub tutorial (https://github.com/smirarab/ASTRAL/blob/master/astral-tutorial.md – last accessed 1/12/2025) for more details on running ASTRAL and the different tree annotation options. This is a useful tutorial for getting familiar with SVDQuartets (https://phylosolutions.com/tutorials/svdq-qage/svdq-qage-tutorial.html – last accessed 1/12/2025) and a tutorial on using PAUP*, which is required to run SVDQuartets (https://paup.phylosolutions.com/tutorials/quick-start/, last accessed 1/12/2025).

1. Use ASTRAL to construct a species tree using multispecies coalescent modelling.

a. First, individual gene trees need to be estimated for the 13 PCGs and 2 rRNAs based on the ML criterion in IQ-Tree using the cleaned and edited single gene alignments created in Section A. Use a greedy model selection strategy (*-m MFP*) and the NNI approach to search for tree topology and compute branch supports with 1000 bootstrapped replicates of the UFBoot2 approach [65].

for gene in *.fas

do

iqtree2 -s $gene -st DNA -m MFP -AICc -nt AUTO -B 1000

done

b. Create a combined file with all the gene tree files.

cat *.treefile > Galeomorphi.15gene.tre

c. Convert the combined treefile to newick format in FigTree (“Galeomorphii-mitophy-15G.tre”).

d. Run the combined gene tree newick file through ASTRAL using the default options.

java -jar astral.5.7.8.jar -i Galeomorphi-mitophy-15G.tre -o Galeomorphi-mitophy-15G-ASTRAL.tre

e. Annotate the branches of the species tree. More details about the different annotation options can be found in the ASTRAL tutorial (https://github.com/smirarab/ASTRAL/blob/master/astral-tutorial.md).

# Construct a fully annotated tree

java -jar astral.5.7.8.jar -q Galeomorphi-mitophy-15G-ASTRAL.tre -i Galeomorphi-mitophy-15G.tre -t 2 -o Galeomorphi-mitophy-15G-scored-t2.tre

# Collapse branches with low support using Newick Utilities and then run ASTRAL.

nw_ed Galeomorphi-mitophy-15G.tre 'i & b<=10' o > Galeomorphi-mitophy-15G-BS10.tre

java -jar astral.5.7.8.jar -i Galeomorphi-mitophy-15G-BS10.tre -o Galeomorphi-mitophy-15G-BS10-ASTRAL.tre

# Generate the posterior probabilities for branches of the main topology and the two alternatives which add up to three for each branch.

java -jar astral.5.7.8.jar -q Galeomorphi-mitophy-15G-ASTRAL.tre -i Galeomorphi-mitophy-15G.tre -t 4 -o Galeomorphi-mitophy-15G-scored-t4.tre

# Show quartet support for the main topology and the two alternative topologies.

java -jar astral.5.7.8.jar -q Galeomorphi-mitophy-15G-ASTRAL.tre -i Galeomorphi-mitophy-15G.tre -t 8 -o Galeomorphi-mitophy-15G-scored-t8.tre

# Test for polytomies with the method of Sayyari and Mirarab 2018, which is based on a Chi-Square test among quartet frequencies for nodes, implemented with the -t 10 command (see Figure 15).

**Figure 15.**
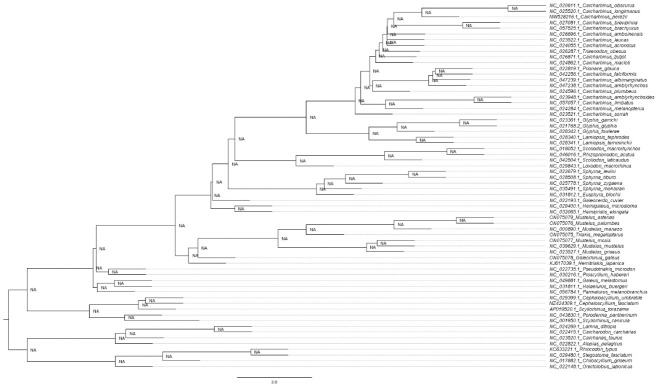
Elasmo-mitophy-15G-scored-t10.tre created to test for polytomy in species in the order Carcharhiniformes (with representative Lamniform and Orectolobiform outgroups) constructed using 13 protein-coding gene trees in ASTRAL v.5.6.3 [49] and visualised in FigTree v.1.4.4 [47]. NA, not applicable, rejects the null hypothesis that the branch is a polytomy.

java -jar astral.5.7.8.jar -q Galeomorphi-mitophy-15G-ASTRAL.tre -i Galeomorphi-mitophy-15G.tre -t `10 -o Galeomorphi-mitophy-15G-scored-t10.tre

f. Visualise the trees in Figtree (Figure 15) or Evolview (see Section C for instructions).

**Figure 16.**
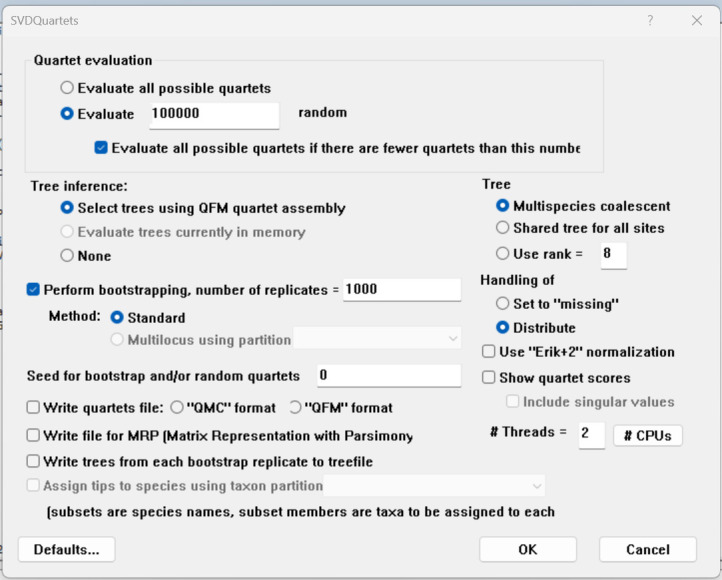
Parameters to select for multispecies coalescent models with SVDQuarters [50] in PAUP* v.4.0a169 [51]. Random quartet sampling of 100,000 replicates and 1,000 bootstrap replicates parameters selected and all other parameters left on default.

**Figure 17.**
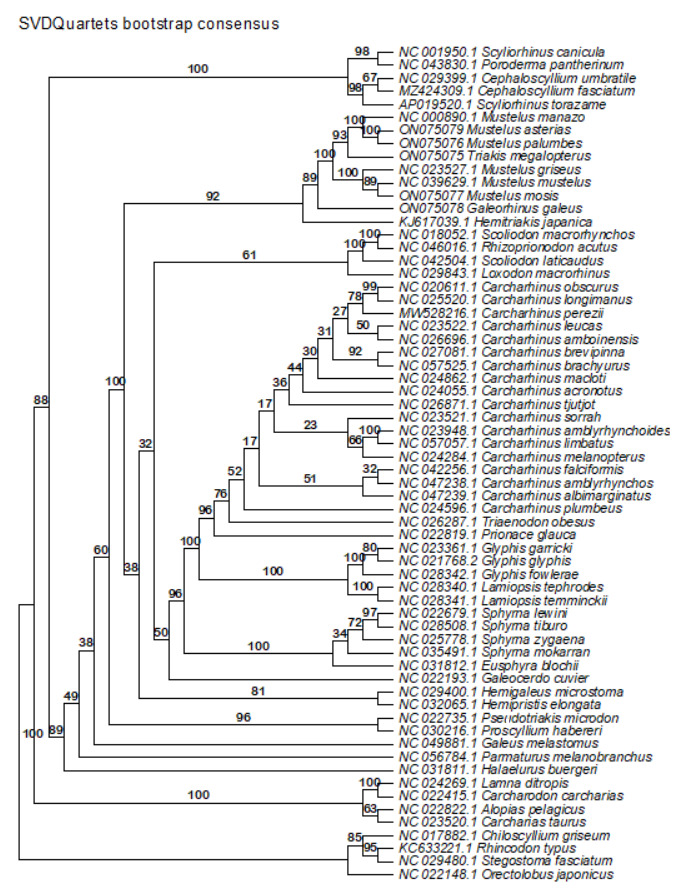
Bootstrap consensus tree for species in the order Carcharhiniformes (with representative Lamniform and Orectolobiform outgroups) constructed using an alignment of the 13 protein-coding genes created using SVDQuarters [50] in PAUP* v.4.0a169 [51]. Bootstrap support values are indicated for each node.

2. Use SVDQuartets to construct a species tree using multispecies coalescent modelling.

a. Create a nexus file with gene partitions using the *Data_17_Galeomorphii_13PCGs_2rRNAs_NT* alignment. This is available as *Data_23_13PCGs_2rRNAs_NT_svd_partitions* (see sample files).

b. Open the nexus file in PAUP*.

c. Implement the multispecies coalescent tree model with random quartet sampling of 100,000 replicates and 1,000 bootstrap replicates (https://phylosolutions.com/tutorials/svdq-qage/svdq-qage-tutorial.html) (Figure 16).

d. Save the bootstrap consensus tree as a png file (Figure 17).

**[Tip 9]** The interpretation of these results is discussed in *General Note 3*, but when viewing these trees ask the following questions:

How different or similar are the gene trees to one another?

How does the topology of the final species tree compare with the ML and BI trees?

Which partitioning strategy yields the most similar topology to the ASTRAL tree?

How does the quartet support for the main topology compare to the two alternative topologies? Are the alternative support values the same or vastly different?

Does the test for polytomy reveal any significant results? Is this expected for the group based on previous literature and divergence knowledge?

How do support values compare for deeper vs. shallower nodes?

**E. Testing site heterogeneous models**

Some datasets may contain evolutionary signatures that violate the assumptions of the applied evolutionary models utilised in traditional phylogenetic reconstruction site-homogenous concatenation-based approaches under maximum likelihood and Bayesian inference frameworks. This requires the application of site-heterogenous models to more accurately elucidate interspecific relationships. However, it is important to first determine whether there is compositional heterogeneity among taxa or among sites in a sequence alignment to decide whether site-heterogenous models are appropriate for the dataset.

Compositional heterogeneity among lineages and across sites can be tested using AliGROOVE v.1.08 [2] and BaCoCa v.1.1 [52]. AliGROOVE is based on a sliding window and a Monte Carlo resampling approach that visualises heterogeneous sequence divergence or alignment ambiguity. The method works by establishing non-random similarity between any two sequences at each site in a matrix of pairwise comparisons relative to the variation in the full set of sequences. BaCoCa assesses base heterogeneity across taxa using the relative composition frequency variability (RCFV) value [52] based on nucleotide or amino acid frequencies.

The protocol described here demonstrates how to conduct analyses in the PHYLOBAYES_MPI v.1.9 package [53] using the GTR + G4 site homogenous model, CAT + Poisson + G4 model (exchange rates are fixed), and the CAT + GTR + G4 model (exchange rates are independently estimated from the data under general time reversible Markov processes). The C60 + GTR + G4 empirical profile mixture model, which provides 60 rate categories to describe rate variation among sites, can be applied to amino acid datasets. The CAT model is a Bayesian site-heterogeneous mixture model [53]. Site-heterogeneous models account for heterogenous equilibrium frequencies across sites by using a Dirichlet process prior to accommodating multiple categories of substitution profiles with diverse nucleotide or amino acid frequencies, along with a single set of exchange rates for the entire supermatrix. The exchange rates can be fixed to flat values (CAT-Poisson) or independently estimated from the data using general time reversible Markov processes (CAT-GTR) [53,73]. All CAT models incorporate a component that accounts for variation in rates across sites, which is set to four by default (G4).

The AliGROOVE (https://github.com/PatrickKueck/AliGROOVE/blob/master/aligroove_gui_howto.pdf, last accessed 1/12/2025) and BaCoCa (https://github.com/PatrickKueck/BaCoCa/blob/master/BaCoCa_Manual.pdf) manuals provide information on setting up and using these programs to test substitutional heterogeneity. Refer to https://github.com/bayesiancook/phylobayes/blob/master/pbManual4.1.pdf (last accessed 1/12/2025) for detailed guidance on running PHYLOBAYES_MPI. Also, see [74] for some important considerations when using CAT models.

**Figure 18.**
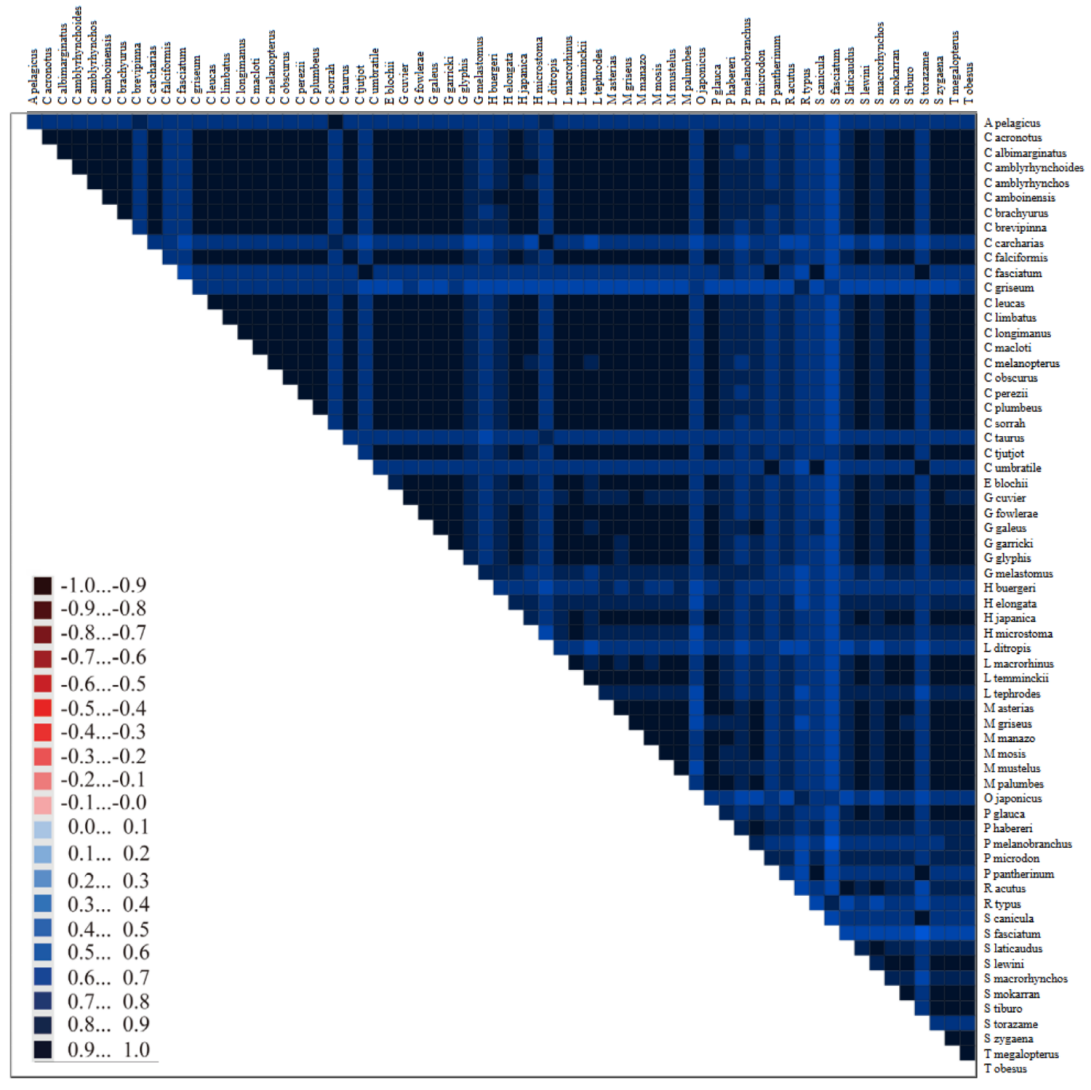
Pairwise comparison of similarity between the mitogenome sequences of Galeomorphii species to test for base compositional heterogeneity in AliGROOVE v.1.08 [2] using Dataset 2: *Data_17_Galeomorphii_13PCGs_2rRNAs_NT.fasta.* Similarity scores are indicated in the key. Positive scores (light blue to dark blue) indicate random similarity due to the descent between pairwise sequence comparisons; negative scores (light pink to maroon) indicate non-random similarity. Either extreme of this scale (-1 and +1) shows up as black but, generally, blocks will show a pattern of blues or reds that will help discern which of the two extremes is present. This plot shows very low heterogeneity amongst lineages in sequence composition for Dataset 2, with all blocks falling in the darker blue range.

1. Use AliGROOVE to test for compositional heterogeneity among lineages in the nucleotide (*Data_14_Galeomorphii_13PCGs_NT.fasta* and *Data_17_Galeomorphii_13PCGs_2rRNAs_NT.fasta*) and amino acid datasets (*Data_20_Galeomorphii_13PCGs_AA.fasta*) (compositional heterogeneity plot for Dataset 2 showed in Figure 18). For nucleotide alignments, we ran the software with the *-N* option to treat indels as ambiguous characters and tested it without invoking *-N* so that indels were treated as a fifth character trait.

aligroove = ./bin/AliGROOVE

# Without invoking the -N option: indels are treated as 5th character

$aligroove/AliGROOVE_v.1.08.pl -i Data_14_Galeomorphii_13PCGs_NT.fasta

$aligroove/AliGROOVE_v.1.08.pl -i Data_17_Galeomorphii_13PCGs_2rRNAs_NT.fasta

$aligroove/AliGROOVE_v.1.08.pl -i Data_20_Galeomorphii_13PCGs_AA.fasta

# With -N option: indels are treated as ambiguous characters (only possible for nucleotide alignments)

$aligroove/AliGROOVE_v.1.08.pl -N -i Data_14_Galeomorphii_13PCGs_NT.fasta

$aligroove/AliGROOVE_v.1.08.pl -N -i Data_17_Galeomorphii_13PCGs_2rRNAs_NT.fasta

2. Run BaCoCa with default settings, invoking the -r option of the program to generate heat maps in combination with hierarchical clustering.

Bacoca = ./BaCoCa_v1.1_Skript_Perl

perl $bacoca/BaCoCa.v1.109.r.pl -i 13PCGs_2rRNAs_NT.fasta -P 13PCGs_2rRNAs_NT.part.txt -r

**Figure 19.**
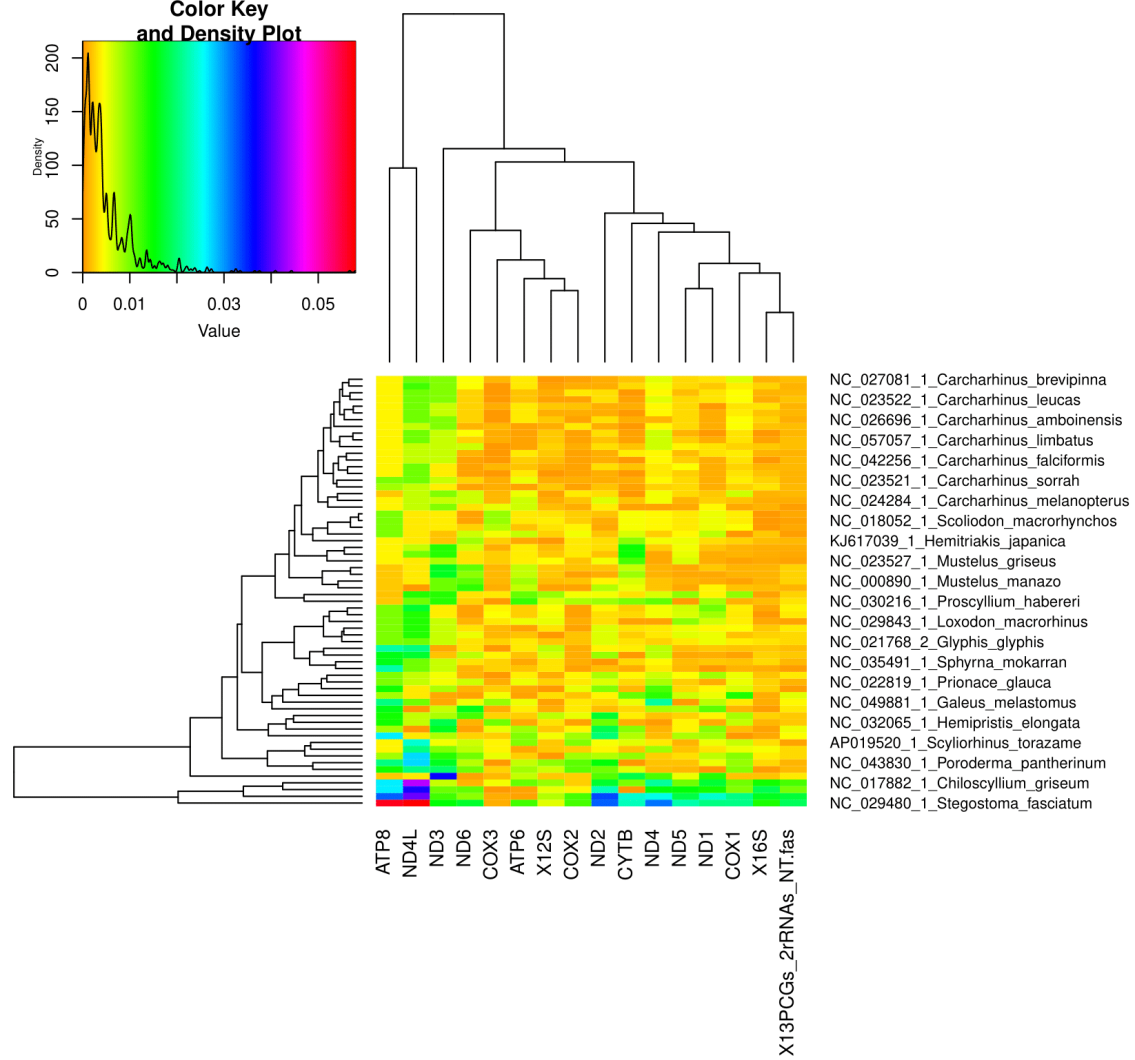
Plot of the compositional heterogeneity of purine characters in the Galeomorphii mitogenome gene sequences created with BaCoCa v.1.1 [52]. Colour density plot shown on the top left (ranging from low to high heterogeneity). This heat map for purine characters shows very low compositional heterogeneity overall, with some outgroup taxa displaying moderate compositional bias.

**[Tip 10]** Refer to the manual https://github.com/PatrickKueck/BaCoCa/blob/master/BaCoCa_Manual.pdf for interpretation of the output tables and figures produced. Figure 19 shows an example of a heat map displaying the compositional heterogeneity of purine characters in the mitogenomes of Dataset 1. We specifically focused on the plots in the folder “compositional bias” to assess whether there was significant compositional heterogeneity among taxa in our dataset for each PCG and the dataset as a whole. The text files in the output folder *chisquare_test_homogeneity_taxa* can be used to produce tables to study compositional heterogeneity in specific taxa.

3. Conduct BI analyses using the *pb_mpi* program of the PHYLOBAYES_MPI package. Run two independent Markov chain Monte Carlo (MCMC) chains per model (GTR + G4, CAT + Poisson + G4, CAT + GTR + G4, and C60 + GTR + G4 for amino acids only) for each supermatrix (*Data_15_Galeomorphii_13PCGs_NT.phy, Data_18_Galeomorphii_13PCGs_2rRNAs_NT.phy, Data_21_Galeomorphii_13PCGs_AA.phy*) for as long as time allows for your project. We ran our chains for several days before checking convergence. Convergence of independent runs can be assessed using maximum difference (*maxdiff*) in all bipartitions and effective sample size (ESS) following the guidelines specified in the PHYLOBAYES_MPI manual (https://github.com/bayesiancook/phylobayes/blob/master/pbManual4.1.pdf). The *bpcomp* program is used to generate outputs of the largest (*maxdiff*) and mean (*meandiff*) discrepancies observed across all bipartitions, while the *tracecomp* program is used to estimate the ESS and discrepancy for the summary variables of the models. For both programs, we used a conservative burn-in of 20% of the length of MCMC chain.

phylobayes = ./data

mod_sel = ./scripts

a. The GTR model

# Dataset 1: 13PCGs_NT

mpirun -np 8 $phylobayes/pb_mpi -ncat 1 -gtr -d Data_15_Galeomorphii_13PCGs_NT.phy GTR_13PCGs_NT_chain1 &

mpirun -np 8 $phylobayes/pb_mpi -ncat 1 -gtr -d Data_15_Galeomorphii_13PCGs_NT.phy

GTR_13PCGs_NT_chain2 &

$phylobayes/tracecomp GTR_13PCGs_NT_chain?.trace -o GTR_13PCGs_NT_tracecomp

$phylobayes/bpcomp -x 813 10 GTR_13PCGs_NT_chain1 GTR_13PCGs_NT_chain2 -o GTR_13PCGs_NT_bpcomp

# Dataset 2: 13PCGs_rRNAs_NT

mpirun -np 8 $phylobayes/pb_mpi -ncat 1 -gtr -d Data_18_Galeomorphii_13PCGs_2rRNAs_NT.phy GTR_13PCGs_2rRNAs_NT_chain1 &

mpirun -np 8 $phylobayes/pb_mpi -ncat 1 -gtr -d Data_18_Galeomorphii_13PCGs_2rRNAs_NT.phy GTR_13PCGs_2rRNAs_NT_chain2 &

$phylobayes/tracecomp GTR_13PCGs_2rRNAs_NT_chain?.trace -o GTR_13PCGs_2rRNAs_NT_tracecomp

$phylobayes/bpcomp -x 764 10 GTR_13PCGs_2rRNAs_NT_chain1 GTR_13PCGs_2rRNAs_NT_chain2 -o GTR_13PCGs_2rRNAs_NT_bpcomp

# *Dataset 3: 13PCGs_AA*

mpirun -np 8 $phylobayes/pb_mpi -ncat 1 -gtr -d Data_21_Galeomorphii_13PCGs_AA.phy GTR_AA_chain1 &

mpirun -np 8 $phylobayes/pb_mpi -ncat 1 -gtr -d Data_21_Galeomorphii_13PCGs_AA.phy GTR_AA_chain2 &

$phylobayes/tracecomp GTR_AA_chain?.trace -o GTR_AA_tracecomp

$phylobayes/bpcomp -x 1373 10 GTR_AA_chain1 GTR_AA_chain2 -o GTR_AA_bpcomp

b. The CAT-Poisson (CAT-F81) model

# Dataset 1: 13PCGs_NT

mpirun -np 24 $phylobayes/pb_mpi -cat -poisson -d Data_15_Galeomorphii_13PCGs_NT.phy CAT-POISSON_13PCGs_NT_chain1 &

mpirun -np 24 $phylobayes/pb_mpi -cat -poisson -d Data_15_Galeomorphii_13PCGs_NT.phy CAT-POISSON_13PCGs_NT_chain2 &

$phylobayes/tracecomp CAT-POISSON_13PCGs_NT_chain?.trace -o CAT-POISSON_13PCGs_NT_tracecomp

$phylobayes/bpcomp -x 2350 10 CAT-POISSON_13PCGs_NT_chain1 CAT-POISSON_13PCGs_NT_chain2 -o CAT-POISSON_13PCGs_NT_bpcomp

# Dataset 2: 13PCGs_2rRNAs_NT

mpirun -np 24 $phylobayes/pb_mpi -cat -poisson -d Data_18_Galeomorphii_13PCGs_2rRNAs_NT.phy CAT-POISSON_13PCGs_2rRNAs_NT_chain1 &

mpirun -np 24 $phylobayes/pb_mpi -cat -poisson -d Data_18_Galeomorphii_13PCGs_2rRNAs_NT.phy CAT-POISSON_13PCGs_2rRNAs_NT_chain2 &

$phylobayes/tracecomp CAT-POISSON_13PCGs_2rRNAs_NT_chain?.trace -o CAT-POISSON_13PCGs_2rRNAs_NT_tracecomp -x 3500

$phylobayes/bpcomp -x 3500 10 CAT-POISSON_13PCGs_2rRNAs_NT_chain1 CAT-POISSON_13PCGs_2rRNAs_NT_chain2 -o CAT-POISSON_13PCGs_2rRNAs_NT_bpcomp

# Dataset 3: 13PCGs_AA

mpirun -np 24 $phylobayes/pb_mpi -cat -poisson -d Data_21_Galeomorphii_13PCGs_AA.phy CAT-POISSON_AA_chain1 &

mpirun -np 24 $phylobayes/pb_mpi -cat -poisson -d Data_21_Galeomorphii_13PCGs_AA.phy CAT-POISSON_AA_chain2 &

$phylobayes/tracecomp CAT-POISSON_AA_chain?.trace -o CAT-POISSON_AA_tracecomp

$phylobayes/bpcomp -x 2601 10 CAT-POISSON_AA_chain1 CAT-POISSON_AA_chain2 -o CAT-POISSON_AA_bpcomp

c. The CAT-GTR model

# Dataset 1: 13PCGs_NT

mpirun -np 24 $phylobayes/pb_mpi -d Data_15_Galeomorphii_13PCGs_NT.phy CAT-GTR_13PCGs_NT_chain1

mpirun -np 24 $phylobayes/pb_mpi -d Data_15_Galeomorphii_13PCGs_NT.phy CAT-GTR_13PCGs_NT_chain2

$phylobayes/tracecomp CAT-GTR_13PCGs_NT_chain1.trace CAT-GTR_13PCGs_NT_chain2.trace -o CAT-GTR_13PCGs_NT_tracecomp

$phylobayes/bpcomp -x 7564 10 CAT-GTR_13PCGs_NT_chain1 CAT-GTR_13PCGs_NT_chain2 -o CAT-GTR_13PCGs_NT_bpcomp

# Dataset 2: 13PCGs_2rRNAs_NT

mpirun -np 24 $phylobayes/pb_mpi -d Data_18_Galeomorphii_13PCGs_2rRNAs_NT.phy CAT-GTR_13PCGs_2rRNAs_NT_chain1

mpirun -np 24 $phylobayes/pb_mpi -d Data_18_Galeomorphii_13PCGs_2rRNAs_NT.phy CAT-GTR_13PCGs_2rRNAs_NT_chain2

$phylobayes/tracecomp CAT-GTR_13PCGs_2rRNAs_NT_chain1.trace CAT-GTR_13PCGs_2rRNAs_NT_chain2.trace -o CAT-GTR_13PCGs_2rRNAs_NT_tracecomp

$phylobayes/bpcomp -x 6170 10 CAT-GTR_13PCGs_2rRNAs_NT_chain1 CAT-GTR_13PCGs_2rRNAs_NT_chain2 -o CAT-GTR_13PCGs_2rRNAs_NT_bpcomp

# Dataset 3: 13PCGs_AA

mpirun -np 24 $phylobayes/pb_mpi -d Data_21_Galeomorphii_13PCGs_AA.phy CAT-GTR_AA_chain1

mpirun -np 24 $phylobayes/pb_mpi -d Data_21_Galeomorphii_13PCGs_AA.phy CAT-GTR_AA_chain2

$phylobayes/tracecomp CAT-GTR_AA_chain1.trace CAT-GTR_AA_chain2.trace -o CAT-GTR_AA_tracecomp

$phylobayes/bpcomp -x 3900 10 CAT-GTR_AA_chain1 CAT-GTR_AA_chain2 -o CAT-GTR_AA_bpcomp

d. C60-GTR model (AA only)

# Dataset 3: 13PCGs_AA

mpirun -np 24 $phylobayes/pb_mpi -catfix c60 -gtr -d Data_21_Galeomorphii_13PCGs_AA.phy C60-GTR_AA_chain1

mpirun -np 24 $phylobayes/pb_mpi -catfix c60 -gtr -d Data_21_Galeomorphii_13PCGs_AA.phy C60-GTR_AA_chain2

$phylobayes/tracecomp C60-GTR_AA_chain?.trace -o C60-GTR_AA_tracecomp

$phylobayes/bpcomp -x 2469 10 C60-GTR_AA_chain1 C60-GTR_AA_chain2 -o C60-GTR_AA_bpcomp

**Figure 20.**
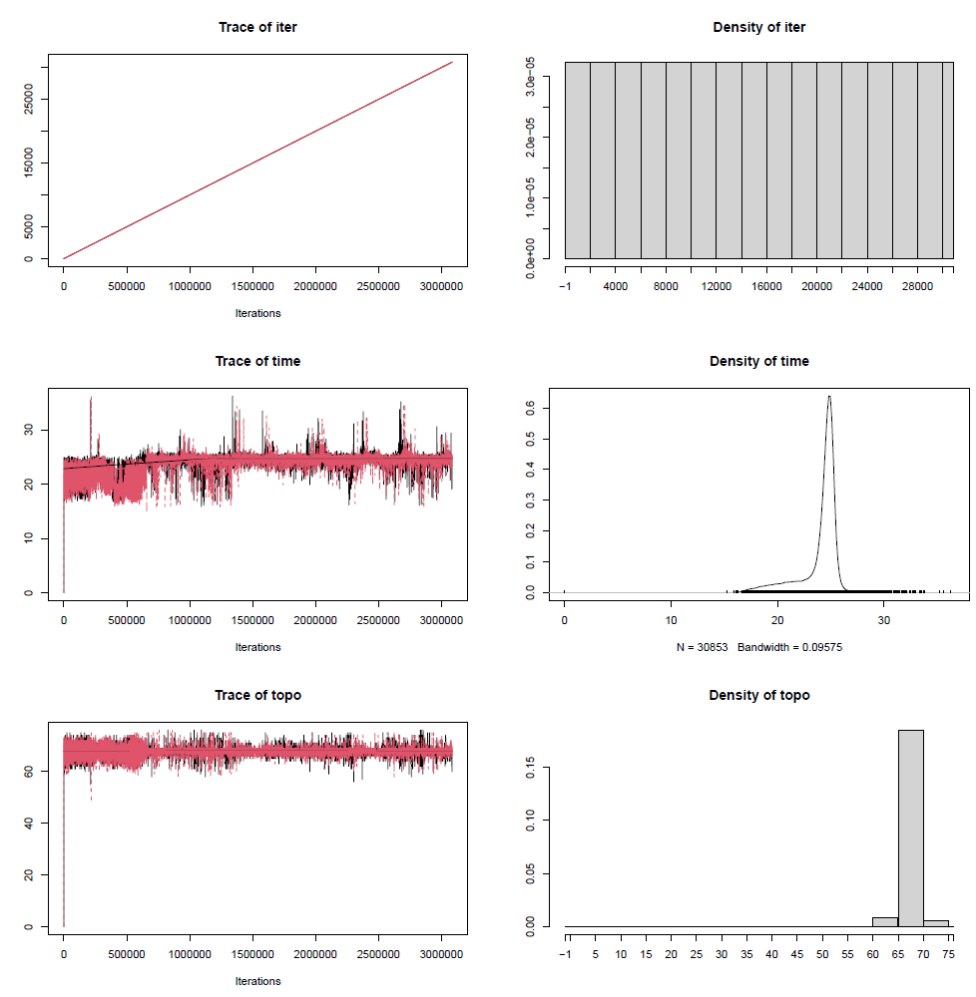
Trace and density plots comparing the number of iterations, run times, and tree topologies of the two chains run under the CAT-GTR model in PHYLOBAYES_MPI v.1.9 [53] for Dataset 2: 13PCGs_2rRNAs_NT in Tracer v.1.7.1 [54]. These tracer plots show good mixing of these parameters for the two chains. The red and black plots representing the two chains begin to converge after ~750,000 iterations.

**Figure 21.**
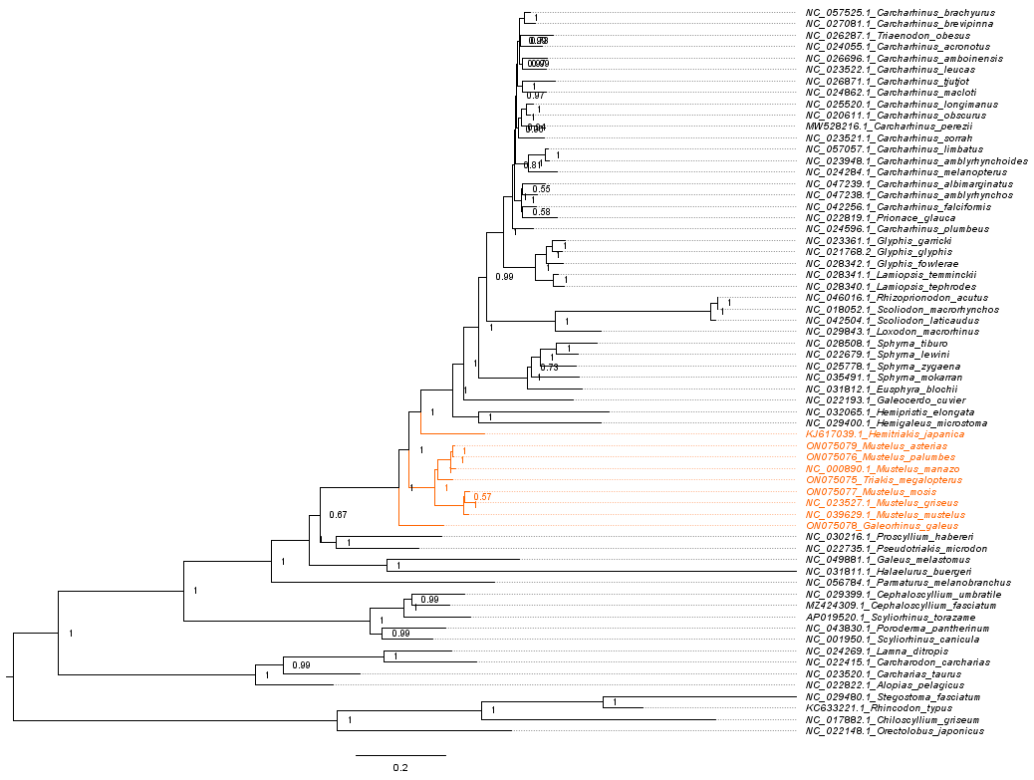
Posterior consensus summary constructed with the site heterogenous CAT+GTR+G4 model for Dataset 2: 13PCGs_2rRNAs_NT in PHYLOBAYES_MPI v.1.9 [63] and visualised in FigTree v.1.4.4 [56]. Posterior probabilities are indicated at the nodes.

4. Check chain convergence in Tracer for the CAT-GTR model. Several output trace and density plots are generated (examples are given in Figure 20). Tutorials with guidance on how to interpret all these plots can be found at https://beast.community/tracer (last accessed 1/12/2025).

5. Visualise and compare the trees for all models generated for each dataset in FigTree (Figure 21).

6. Generate site-specific log-likelihood statistics for the different models with the *readpb_mpi* program. Use a burn-in of 20% of the length of the MCMC chain and thin accordingly to obtain a total of at least 100 MCMC points given the computational intensiveness of estimating site log-likelihoods under the CAT models.

phylobayes = ./data

# Dataset 1: 13PCGs_NT

mpirun -np 48 $phylobayes/readpb_mpi -x 813 32 -sitelogl GTR_13PCGs_NT_chain1

mpirun -np 48 $phylobayes/readpb_mpi -x 813 32 -sitelogl GTR_13PCGs_NT_chain2

mpirun -np 48 $phylobayes/readpb_mpi -x 2350 94 -sitelogl CAT-POISSON_13PCGs_NT_chain1

mpirun -np 48 $phylobayes/readpb_mpi -x 2350 94 -sitelogl CAT-POISSON_13PCGs_NT_chain2

mpirun -np 48 $phylobayes/readpb_mpi -x 7564 300 -sitelogl CAT-GTR_13PCGs_NT_chain1

mpirun -np 48 $phylobayes/readpb_mpi -x 7564 300 -sitelogl CAT-GTR_13PCGs_NT_chain2

# Dataset 2: 13PCGs_rRNAs_NT

mpirun -np 48 $phylobayes/readpb_mpi -x 764 30 -sitelogl GTR_13PCGs_2rRNAs_NT_chain1

mpirun -np 48 $phylobayes/readpb_mpi -x 764 30 -sitelogl GTR_13PCGs_2rRNAs_NT_chain2

mpirun -np 48 $phylobayes/readpb_mpi -x 3500 47 -sitelogl CAT-POISSON_13PCGs_2rRNAs_NT_chain1

mpirun -np 48 $phylobayes/readpb_mpi -x 3500 47 -sitelogl CAT-POISSON_13PCGs_2rRNAs_NT_chain2

mpirun -np 48 $phylobayes/readpb_mpi -x 6170 242 -sitelogl CAT-GTR_13PCGs_2rRNAs_NT_chain1

mpirun -np 48 $phylobayes/readpb_mpi -x 6170 242 -sitelogl CAT-GTR_13PCGs_2rRNAs_NT_chain2

# Dataset 3: 13PCGs_AA

mpirun -np 48 $phylobayes/readpb_mpi -x 1373 54 -sitelogl GTR_AA_chain1

mpirun -np 48 $phylobayes/readpb_mpi -x 1373 54 -sitelogl GTR_AA_chain

mpirun -np 48 $phylobayes/readpb_mpi -x 2469 98 -sitelogl C60-GTR_AA_chain1

mpirun -np 48 $phylobayes/readpb_mpi -x 2469 98 -sitelogl C60-GTR_AA_chain2

mpirun -np 48 $phylobayes/readpb_mpi -x 2601 104 -sitelogl CAT-POISSON_AA_chain1

mpirun -np 48 $phylobayes/readpb_mpi -x 2601 104 -sitelogl CAT-POISSON_AA_chain2

mpirun -np 48 $phylobayes/readpb_mpi -x 3900 154 -sitelogl CAT-GTR_AA_chain1

mpirun -np 48 $phylobayes/readpb_mpi -x 3900 154 -sitelogl CAT-GTR_AA_chain2

a. Collect the site-specific scores.

# Dataset 1: 13PCGs_NT

python3 ../scripts/read_loocv_waic.py GTR_13PCGs_NT_chain?.sitelogl

python3 ../scripts/read_loocv_waic.py CAT-POISSON_13PCGs_NT_chain?.sitelogl

python3 ../scripts/read_loocv_waic.py CAT-GTR_13PCGs_NT_chain?.sitelogl

# Dataset 2: 13PCGs_rRNAs_NT

python3 ../scripts/read_loocv_waic.py GTR_13PCGs_2rRNAs_NT_chain?.sitelogl

python3 ../scripts/read_loocv_waic.py CAT-POISSON_13PCGs_2rRNAs_NT_chain?.sitelogl

python3 ../scripts/read_loocv_waic.py CAT-GTR_13PCGs_2rRNAs_NT_chain?.sitelogl

# Dataset 3: 13PCGs_AA

python3 ../scripts/read_loocv_waic.py GTR_AA_chain?.sitelogl

python3 ../scripts/read_loocv_waic.py CAT-POISSON_AA_chain?.sitelogl

python3 ../scripts/read_loocv_waic.py C60-GTR_AA_chain?.sitelogl

python3 ../scripts/read_loocv_waic.py CAT-GTR_AA_chain?.sitelogl

b. Generate scores for the widely applicable information criterion (wAIC) and the asymptotically equivalent leave one-out cross-validation (LOO-CV) [75] using the script *read_loocv_waic.py*, which is provided as part of the PHYLOBAYES_MPI package.

# Load the package

library(coda)

# Read the data

chain1 <- read.table("CAT-GTR_AA_chain1_reduced2matchchanin2.trace",header=TRUE)

chain2 <- read.table("CAT-GTR_AA_chain2.trace",header=TRUE)

chain3 <- read.table("CAT-GTR_13PCGs_NT_chain1.trace",header=TRUE)

chain4 <- read.table("CAT-GTR_13PCGs_NT_chain2_reduced2matchchanin1.trace",header=TRUE)

chain5 <- read.table("CAT-GTR_13PCGs_2rRNAs_NT_chain1.trace",header=TRUE)

chain6 <- read.table("CAT-GTR_13PCGs_2rRNAs_NT_chain2_reduced2matchchanin1.trace",header=TRUE)

# Create an MCMC object with the correct thinning interval (used 1000).

chain1 <- mcmc(chain1,thin=100)

chain2 <- mcmc(chain2,thin=100)

chain3 <- mcmc(chain3,thin=100)

chain4 <- mcmc(chain4,thin=100)

chain5 <- mcmc(chain5,thin=100)

chain6 <- mcmc(chain6,thin=100)

# Always start with a plot. A rough way of verifying convergence is to plot the trace and the posterior distribution of some of the parameters.

pdf("CAT-GTR_AA_chain1.trace.plot.pdf", width=10, height=10)

plot(chain1)

.=dev.off()

pdf("CAT-GTR_AA_chain2.trace.plot.pdf", width=10, height=10)

plot(chain2)

.=dev.off()

pdf("CAT-GTR_13PCGs_NT_chain1.trace.plot.pdf", width=10, height=10)

plot(chain3)

.=dev.off()

pdf("CAT-GTR_13PCGs_NT_chain2.trace.plot.pdf", width=10, height=10)

plot(chain5)

.=dev.off()

pdf("CAT-GTR_13PCGs_2rRNAs_NT_chain1.trace.plot.pdf", width=10, height=10)

plot(chain5)

.=dev.off()

pdf("CAT-GTR_13PCGs_2rRNAs_NT_chain2.trace.plot.pdf", width=10, height=10)

plot(chain6)

.=dev.off()

# Obtaining summary statistics for the MCMC chain.

summary(chain1)

summary(chain2)

summary(chain3)

summary(chain4)

summary(chain5)

summary(chain6)

# Is the sample from the MCMC large enough?

autocorr.diag(chain1)

effectiveSize(chain1)

autocorr.diag(chain2)

effectiveSize(chain2)

autocorr.diag(chain3)

effectiveSize(chain3)

autocorr.diag(chain4)

effectiveSize(chain4)

autocorr.diag(chain5)

effectiveSize(chain5)

autocorr.diag(chain6)

effectiveSize(chain6)

# Has the chain converged?

combined.d1 = mcmc.list(chain1,chain2)

pdf("CAT-GTR_AA_chains-combined.trace.plot.pdf", width=10, height=10)

plot(combined.d1)

.=dev.off()

gelman.diag(combined.d1)

pdf("CAT-GTR_AA_chains-combined.trace.gelmanplot.pdf", width=10, height=10)

gelman.plot(combined.d1)

.=dev.off()

combined.d2 = mcmc.list(chain3,chain4)

pdf("CAT-GTR_13PCGs_NT_chains-combined.trace.plot.pdf", width=10, height=10)

plot(combined.d2)

.=dev.off()

gelman.diag(combined.d2)

pdf("CAT-GTR_13PCGs_NT_chains-combined.trace.gelmanplot.pdf", width=10, height=10)

gelman.plot(combined.d2)

.=dev.off()

combined.d3 = mcmc.list(chain5,chain6)

pdf("CAT-GTR_13PCGs_2rRNAs_NT_chains-combined.trace.plot.pdf", width=10, height=10)

plot(combined.d3)

.=dev.off()

gelman.diag(combined.d3)

pdf("CAT-GTR_13PCGs_2rRNAs_NT_chains-combined.trace.gelmanplot.pdf", width=10, height=10)

gelman.plot(combined.d3)

.=dev.off()

7. Compare the different models for each dataset by constructing a table containing the convergence statistics and model comparison scores generated. Table 7 shows the table we constructed for Dataset 2.

**Table 7.** Bayesian posterior comparison (*bcomp*) and trace comparison (*tracecomp)* output statistics to assess convergence of the two chains run for each model for Dataset 2 (*13PCGs_2rRNAs_NT*) with the *readpb_mpi* program of PHYLOBAYES_MPI v.1.9 [53]. Burn-in was set to 20% of the length of Markov chain Monte Carlo (MCMC) chain.

Model	GTR + G4		CAT + Poisson + G4		CAT + GTR + G4	
***bcomp*-stats**	Chain 1	Chain 2	Chain 1	Chain 2	Chain 1	Chain 2
*number of trees*	305	307	472	490	2468	2500
*maxdiff*	0.087286		0.294552		0.052572	
*meandiff*	0.001040		0.010465		0.002457	
***tracecomp*-stats**	Chain 1	Chain 2	Chain 1	Chain 2	Chain 1	Chain 2
*upper limit*	3822		8227		30853	
*burnin*	764	764	3500	3500	6170	6170
*sample size*	3058	3058	4727	4727	24683	24683
*name*	***effsize***	***rel_diff***	***effsize***	***rel_diff***	***effsize***	***rel_diff***
*loglik*	3058	0.022587	246	0.059391	2166	0.039804
*length*	2061	0.037407	2440	0.098888	4745	0.020185
*alpha*	272	0.054673	373	0.051591	2108	0.022192
*Nmode*	3058	0.000000	175	0.035989	3980	0.014003
*statent*	964	0.059726	552	0.021961	2991	0.035832
*statalpha*	3058	0.000000	175	0.042154	2646	0.057976
*rrent*	904	0.016250			14335	0.015774
*rrmean*	206	0.073493			6315	0.015618
***readpb*-parameters**	Chain 1	Chain 2	Chain 1	Chain 2	Chain 1	Chain 2
*burnin*	764	764	3500	3500	6170	6170
*thinning*	30	30	47	47	242	242
Target MCMC	101	101	100	100	101	101
***read_loocv_waic-stats***	**LOO-CV**	**waic**	**LOO-CV**	**waic**	**LOO-CV**	**waic**
*debiased score*	-15.078	-15.078	-14.971	-14.971	-14.555	-14.554
*bias*	0.000	0.000	0.000	0.000	0.000	0.000
*stdev*	0.000	0.000	0.000	0.000	0.000	0.000
*CI95min*	-15.078	-15.079	-14.972	-14.973	-14.557	-14.558
*CI95max*	-15.078	-15.077	-14.970	-14.968	-14.552	-14.550
*ess*	101.296	101.291	100.925	100.980	100.947	100.993
*%(ess<10)*	0.000	0.000	0.000	0.000	0.000	0.000
*f(ess<10)*	0.000	0.000	0.000	0.000	0.000	0.000
Triakidae grouping	Monophyletic		Paraphyletic		Paraphyletic	

Table 8 shows the average value of sampled points from the posterior distribution obtained via MCMC for the different models. These should be consistent across different thinning values if the MCMC chain has properly converged and the effective sample size is sufficient. Large variations in MCMC estimates might indicate that the chain has not fully converged, thinning is too aggressive and is reducing sample diversity, or the effective sample size is too small, leading to unreliable estimates.

**Table 8.** Estimated means of the posterior samples under different thinning values for the Markov chain Monte Carlo (MCMC) chains run for each model of Dataset 2 (*13PCGs_2rRNAs_NT*) during analyses with the *readpb_mpi* program of PHYLOBAYES_MPI v.1.9 [53].

	GTR + G4	CAT + Poisson + G4	CAT + GTR + G4
*Thinning*	30	47	242
*MCMC*	101.9333	100.5745	101.9959

**Maxdiff:** Maximum bipartition difference between posterior probabilities of the chains (values below 0.1 are considered to indicate good chain convergence).

**Meandiff:** Mean bipartition difference between posterior probabilities of the chains.

**Effsize:** Effective sample size of the bipartition support (reflects how much information contributes to the posterior probability of a particular bipartition in the phylogenetic tree).

**Reldiff:** Relative difference in posterior probabilities for bipartitions between two independent MCMC chains. A small reldiff value (typically close to 0) suggests that the posterior probabilities for the bipartition are very similar between the two chains, indicating good convergence and consistency.

**Loglik:** Log-likelihood score.

**Alpha:** Shape parameter of the gamma distribution used to model rate heterogeneity across sites.

**Nmode:** Number of distinct substitution categories inferred from the data.

**Statent:** Stationary entropy (diversity of stationary probabilities across sites).

**Statalpha:** Dirichlet process concentration parameter (controls the granularity of site-specific substitution profiles).

**Rrent:** Relative rate entropy (variability in substitution rates across sites).

**Rrmean:** Mean of the relative rates across sites (reflects the rate of substitution at each site compared to a baseline or average rate across all sites).

**Read_loocv_waic-stats:** Read Leave-One-Out Cross-Validation and Widely Applicable Information Criterion (WAIC) statistics.

**Debiased score:** Evaluates the fit of a model to the data by correcting for certain biases that might affect the estimation of the model parameters.

**Stdev:** Standard deviation.

**C195min and max:** Threshold for minimum and maximum effective sample size that a parameter must reach to converge.

**Ess:** Effective sample size

**%(eff<10):** Percentage of ESS that is less than 10

**f(ess<10):** Fraction of the ESS that is less than 10

**[Tip 11]** Look at the weighted AIC scores for each model. Here, they indicate that the complex CAT + GTR + G4 model best fits this dataset (it has the lowest wAIC). According to guidelines relating to the convergence of independent runs, maxdiff must be below 0.1, the effsize for each parameter must be greater than 100, and the rel_diff of parameters among chains must be below 0.1. Also, take note of the resulting topological outcome for each model. In Dataset 2, the triakids were recovered as paraphyletic for the site heterogeneous models and monophyletic with the site homogenous GTR + G4 model. For the triakids, the effective sample size values for the entropy of the relative exchange rates (rrent) and posterior means of the exchange abilities parameter (rrmeans) are consistently high across chains and datasets. Higher values for a specific pair of character states indicate a higher rate of substitution between those states, implying that solely the GTR component of the CAT + GTR + G4 model is reliably converging across datasets, particularly with respect to Datasets 2 and 3. Furthermore, the failure of our amino acid dataset (Dataset 3) to converge after over 19,500 MCMC suggests that the chosen model may not accurately represent the underlying evolutionary processes in the amino acid dataset. See Troubleshooting and General tips for more advice on how to interpret these results.

## Validation of protocol

This full protocol has been used and validated in the following research article:

• Winn et al. [83]. A comprehensive phylogenomic study unveils evolutionary patterns and challenges in the mitochondrial genomes of Carcharhiniformes: A focus on Triakidae. *Genomics*. 116(1): 110771. doi: 10.1016/j.ygeno.2023.110771

An extensive statistical workflow was used to expand the phylogeny of Triakidae. We began by retrieving representative mitogenomes from the Carcharhiniform order, including four outgroups each from the Lamniform and Orectolobiform orders, and merged the GenBank records with our newly assembled mitogenomes as per the sequence alignment and concatenation pipeline of Section A (these mitogenomes are available as Data 13 on Dryad). Hereafter, we extracted protein-coding genes (PCGs) and ribosomal (r)RNAs, manually standardised gene names, extracted and orientated individual genes in the correct order, produced codon-aware multiple sequence alignments for each of the 13 PCGs using MACSE and the rRNAs using MAFFT with the Q-INS-i algorithm, manually trimmed the alignments in Geneious, and removed any remaining ambiguously aligned sites using BMGE. The gene alignments were used to produce three concatenated mitogenomic datasets: Dataset 1: 13PCGs_NT dataset; Dataset 2: 13PCGs_rRNAs_NT; Dataset 3: 13PCGs_AA dataset (available as Data 14–23 on Dryad). Length summaries were saved to use for partition file construction.

In Section B, we performed two-tailed tests of substitution saturation [56] for each gene and each codon position of the 13PCGs as well as on the entire 13PCGs_NT and 13PCGs_rRNAs_NT datasets in DAMBE [42,43]. We also plotted the number of transitions (s) and transversions (v) vs. divergence to visually inspect substitution saturation. These results were used to inform the selection of 10 partition nexus files, two for dataset 1, six for dataset 2, and two for dataset 3 (available in the *4_Partition_Files* folder on Dryad). We found little evidence of substitution saturation across genes and codon positions (Figure 8 and Table 1), so none were excluded from partitioning strategies. However, codon position 2 was found to add little phylogenetic informativity, and hence, we tested some partitioning schemes excluding codon 2.

As per the pipeline for mitophylogenomic reconstruction, the 10 partition files (Table 2) were each used as a priori input into ModelFinder in IQ-Tree to determine the best partitioning scheme and corresponding evolutionary models for maximum likelihood analysis. We compared bootstrap values and conducted an approximately unbiased (AU) tree topology test [59] in IQ-Tree to determine how the choice in partitioning strategy influenced phylogenetic inference. Secondary model selection in IQ-Tree was conducted to select the next best model for Bayesian inference, which was performed using MrBayes. Additionally, we investigated topological conflict around each branch of the species tree by calculating gene (gCF) and site concordance factors (sCF) in IQ-Tree. We then used a χ2-test to see if the frequency of gene trees (gCF) and sites (sCF) supporting the two alternative topologies differed significantly as implemented in Lanfear’s R script [60] (Table 6). We also constructed plots comparing gCF and sCF values with UFBoot2 values using Lanfear’s R script (Figures 9 and 10).

Deeper nodes of the tree were consistent and resolved with high confidence across partition schemes, indicating that the phylogenetic signal in our dataset was robust (Figures 11–14 here and Figure 5 in the original research article). However, discordance was evident for more recently diverged lineages, particularly within the Carcharhinidae and Triakidae families. This is likely the consequence of incomplete lineage sorting, where not enough time has passed to sort out ancestral genetic variation in immediate descendants. In this study, the best supported partitioning schemes were PS4 (codon: pos1 + pos3 + 2rRNAs) and PS8 (geneXcodon: 13 PCGs pos1 + pos3 + 2rRNAs). However, they divide the dataset up extensively, likely reducing the signal available to resolve lineages experiencing incomplete lineage sorting (ILS) and leading to the overparameterisation of model parameters and inflation of bootstrap support values. Concordance factors were low for shallower nodes across partitioning schemes, but particularly for geneXcodon-partitioned phylogenies (PS06–PS08) where there are a high number of partitions. gCFs were lower than sCFs, pointing toward gene tree discordance. Chi-squared tests confirmed that the frequencies of gene and site trees supporting alternative topologies of gene tree quartets were comparable, confirming that ILS was present in our dataset (Table 6).

With dataset partitioning under ML or BI frameworks yielding contrasting resolutions for shallower lineages due to ILS, we employed the summary method, ASTRAL, and the site-based method, SVDQuartets, to estimate the effects of gene-tree conflict on species-tree inference under the multispecies coalescent model (Figures 15 and 17 and Figure 6 in the original research article). The species trees constructed resulted in monophyletic clustering of Triakidae. The genus *Carcharhinus* still showed paraphyly and variation across ML, BI, and MSCM topologies, suggesting that not enough time has passed to confidently resolve some of the relationships between species that have undergone recent rapid radiations.

Lastly, we tested for compositional heterogeneity in the dataset using AliGROOVE and BaCoCa (Figures 18 and 19) and then conducted Bayesian inference analyses using the pb_mpi program in the PHYLOBAYES_MPI package to test a site homogenous model (GTR + G4), a site heterogeneous model with fixed exchange rates (CAT + Poisson + G4), and a site heterogenous model where exchange rates are independently estimated from the data under general time reversible Markov processes (CAT + GTR + G4). The C60 + GTR + G4 empirical profile mixture model, which provides 60 rate categories to describe rate variation among sites, was applied to our amino acid datasets. It was discovered that compositional heterogeneity was low for this dataset, and site heterogenous models were not appropriate because they overestimated the number of substitutional categories in the dataset, resulting in overparameterisation (Table 7).

## General notes and troubleshooting

**General notes**

1. Selecting ingroup and outgroup taxa

In Section A, we provide our ingroup and outgroup sampling strategy as well as code to enable more efficient gene extraction, alignment, cleaning, and concatenation to create alignment datasets for phylogenetic investigations. The phylogenomic dataset selected must be carefully selected and curated to include sufficient representation of all taxa and appropriate outgroups. For example, if you are reconstructing the phylogenetic tree for an order, representatives from each family belonging to the order should be included where possible. Outgroup selection must also be carefully considered. Smith [76] recommends sampling the two closest successive sister groups of the ingroup clade when selecting the outgroups. When sampling only a single outgroup, it is not possible to test the monophyly of the ingroup [77,78].

2. Assessing evolutionary patterns of a dataset to select partitioning schemes

Before constructing phylogenetic trees, data partitioning is necessary for concatenation-based maximum likelihood and Bayesian inference phylogenetic reconstruction. Our data partitioning strategy was carefully curated and selected based on our investigation of the evolutionary signals in our dataset. We tested for substitution saturation in Section B. Saturation occurs when multiple substitutions have occurred at the same site, compromising the signal in the dataset, and making it difficult to accurately infer the true historical relationships between sequences. A gene or codon position that is saturated could be omitted to improve the reliability of phylogenetic analyses. For example, if codon position 3 possesses a high degree of substitution saturation across the PCGs and the entire alignment dataset, it can be excluded from a priori partitioning schemes.

Figure 9 shows a graphical assessment of the number of transitions (s) and transversions (v) on the y-axis vs. divergence on the x-axis for ND1. If the plot reaches a plateau, it suggests that further divergence does not lead to a proportional increase in substitutions; however, different regions of the genome or different organisms may naturally exhibit variations in s/v ratios. Additionally, factors like codon usage, selection pressures, and the specific mutational context can all influence the observed ratio. It is important to consider the ratios in the context of the specific research question and the biology of the sequences under investigation. In our analyses, very little substitution saturation was observed across genes and codon positions, so all PCGs could be included in phylogenetic analyses. Codon position two (pos_2) contained low informativity across many of the genes. The plot for pos_2 of ND1 (and all our other genes) is relatively scattered around the diagonal and combining pos_1 and pos_2 yields a similar outcome to pos_1 alone, indicating that pos_2 may not provide much additional information for phylogenetic purposes. Accordingly, we omitted pos_2 from a priori partitioning strategy 4 (PS04) and PS08 and investigated how this influenced phylogenetic reconstruction (Table 2).

3. Selecting an appropriate mitophylogenomic reconstruction approach

Sections C–E provide an extensive bioinformatic process for mitophylogenomic reconstruction. There is no “one-size-fits-all” approach to mitophylogenomics, and it is paramount to carefully evaluate the evolutionary trends present in your own dataset before selecting the best strategy to follow (refer to Figure 1). If your dataset has sufficient signal (the clustering pattern of main groups is consistent across partitioning strategies, with high support values for deeper nodes) and does not show signs of incomplete lineage sorting or compositional heterogeneity, applying ML and BI approaches to a partitioned concatenated alignment may be sufficient. However, when there is variation in evolutionary rate amongst genes and/or sites, the a priori partitioning strategy applied to the dataset can have a considerable influence on the phylogenetic outcome. In Section C, we detailed how to compare different a priori partitioning strategies in ML and BI analyses. On a first evaluation, the evolutionary models and partitioning strategies produced using a priori schemes with the highest log likelihood scores and lowest AICc and BIC scores best fit the dataset (Table 3). However, it is important to be aware that complex models with many partitions may lead to overfitting, where the model fits the noise in the data rather than capturing the true underlying patterns [23,28,29]. This means that, regardless of the suitability of a given partitioning strategy for the dataset, resampling may always return the same tree (i.e., 100% bootstrap support), even though incomplete lineage sorting or other processes that lead to genealogical discordance are at work [60]. This becomes apparent after a comparison of the concordance factors with bootstrap values in Figure 9, where bootstrap values are high even when gCF and sCF values are low. When gCF values are higher than sCF values (as they are for the deeper nodes of our tree; Figure 12), it indicates that sufficient phylogenetic signal exists in the dataset because these nodes are always resolved with high confidence. However, when gCF values are low, as they are for more recently diverged lineages of our ML topology that have shorter branches, this is an artifact of discordance among gene trees. Low sCFs may indicate that there is discordance between individual genomic sites and the inferred species tree, or certain relationships among lineages are not well-resolved because divergence happened very recently or there is genuine polytomy. Polytomy can be tested in ASTRAL as described in Section D. After conducting Chi-squared tests to determine whether the frequencies of gCF and sCF supporting the two alternative topologies differed significantly and obtaining non-significant p-values (p > 0.05) (Table 6), it was determined that discordance among gene trees and/or sites across lineages is most likely due to incomplete lineage sorting. This meant that deploying multispecies coalescent models was the most appropriate solution for our dataset (Section D).

If the frequencies of gCF and sCF for the alternative topologies do differ significantly, this may be indicative of unequal rates of evolution of genes among different lineages if hybridisation can be ruled out for your study group. In this instance, the use of site heterogeneous models, as described in Section E, would be the preferred next step. Site-heterogeneous models can be used to address heterogeneity in substitution rates across sites, across lineages, and/or among sites. The accuracy of findings from Bayesian phylogenetic analyses requires the chains to have reached a state of stability, known as stationarity, and obtaining a substantial number of independent samples from the posterior distribution. However, many studies that have applied site-heterogeneous models have experienced difficulties in achieving acceptable metrics of convergence, even after running the chains for years [74,79–81]. The failure of chains to converge suggests a chosen model may not accurately represent the underlying evolutionary processes occurring in a dataset. When the CAT model is applied to a dataset without compositional heterogeneity, other information may be captured, because CAT models are theoretically infinitely complex and may overestimate the number of substitutional categories in the dataset [74,82]. This highlights the importance of taking a careful approach when selecting appropriate models and even phylogenetic approaches for a given dataset. We recommend testing for compositional heterogeneity using AliGROOVE and BaCoCa before electing to dedicate computational time and resources to these models. While site heterogenous models in PHYLOBAYES_MPI may be effective in handling site heterogeneity and mitigating long branch attraction, they do not consider gene boundaries, and it is therefore not regarded as an appropriate approach for addressing incomplete lineage sorting as seen in our dataset.

**Troubleshooting**

**Problem 1:** Discordance at deep nodes of the trees.

**Possible causes:** There is low informativity in the dataset or branch length heterogeneity/long-branch attraction (violation of stationarity).

**Solution:** Consider adding additional available mitogenomes across representative taxa.

**Problem 2:** Chains are not converging in PHYLOBAYES_MPI.

**Possible causes:** Insufficient chain length (chains have not been run long enough to sample the posterior distribution adequately), mismatch between priors and the dataset (if the priors on parameters like branch lengths and substitution rates are not appropriate for the dataset, this can lead to chains getting stuck in non-optimal regions of the parameter space or slow convergence), inappropriate or complex models (the chosen evolutionary model may not be appropriate for the dataset, or the model may be too complex, leading to poor mixing or overparameterisation).

**Solution:** Use more chains (increase the number of independent chains) and increase the length of burn-in to allow chains more time to explore the parameter space. Adjust prior settings to make them more flexible, allowing for better parameter space exploration. Some datasets may require more flexible models to improve mixing (e.g., the CAT-Poisson model instead of CAT-GTR). Alternatively, if you are using a simple model and the data is highly heterogeneous, try using more complex models that better capture the data (e.g., CAT models). Also, try relaxing constraints on model parameters when using the CAT model in case PHYLOBAYES_MPI underestimates the number of site categories. Always check the alignment quality and consider removing problematic sequences or sites. Use tracecomp and bpcomp to monitor convergence and adjust the run, as necessary.

**Problem 3:** Issues running a code or software program

**Solution:** Consult the manual for the relevant software to determine potential issues. Here are some useful resources:

https://omicstutorials.com/comprehensive-guide-to-setting-up-and-using-linux-for-bioinformatics-analysis/ (last accessed 1/12/2025): Comprehensive Guide to Setting up and Using Linux for Bioinformatics Analysis.

http://dambe.bio.uottawa.ca/publications/2009PhylHandbookChap20.pdf (last accessed 1/12/2025): Chapter from “The Phylogenetic Handbook” on assessing substitution saturation with DAMBE.

http://www.iqtree.org/doc/ (last accessed 1/12/2025): IQ-Tree manual with detailed instructions for how to select parameters and run the program as well as output file descriptions.

https://www.robertlanfear.com/blog/files/concordance_factors.html (last accessed 1/12/2025): Script and tutorial for conducting χ2-tests to compare concordance factors and bootstrap values.

https://pmc.ncbi.nlm.nih.gov/articles/PMC5624502/ (last accessed 1/12/2025): A biologist’s guide to Bayesian phylogenetic analysis.

https://github.com/smirarab/ASTRAL/blob/master/astral-tutorial.md (last accessed 1/12/2025): Detail for running ASTRAL and tree annotation options.

https://phylosolutions.com/tutorials/svdq-qage/svdq-qage-tutorial.html (last accessed 1/12/2025): Tutorial for running SVDQuartets.

https://paup.phylosolutions.com/tutorials/quick-start/ (last accessed 1/12/2025): Quick start guide to using PAUP*, which is required to run SVDQuartets.

https://github.com/PatrickKueck/AliGROOVE/blob/master/aligroove_gui_howto.pdf (last accessed 1/12/2025): AliGROOVE manual.

BaCoCa (https://github.com/PatrickKueck/BaCoCa/blob/master/BaCoCa_Manual.pdf (last accessed 1/12/2025): BaCoCA manual.

https://github.com/bayesiancook/phylobayes/blob/master/pbManual4.1.pdf (last accessed 1/12/2025): Guidelines for running PHYLOBAYES_MPI. Also, see [74] for some important considerations when using CAT models.
